# Frequency of Consumption of Food Groups and Cardio-Metabolic Risk Factors: A Genetically Informative Twin Study in Sri Lanka

**DOI:** 10.1007/s10519-023-10165-8

**Published:** 2023-12-22

**Authors:** Helena M. S. Zavos, Laura Riddleston, Kaushalya Jayaweera, Lasith Dissanayake, Sameeha Jabir, Gayani Pannala, Matthew Hotopf, Sisira Siribaddana, Athula Sumathipala, Frühling V. Rijsdijk

**Affiliations:** 1 Department of Psychology, Institute of Psychiatry, Psychology and Neuroscience, King’s College London, London, UK; 2 Youth Resilience Unit, Wolfson Institute of Population Health, Queen Mary University of London, London, UK; 3 Institute for Research and Development in Health and Social Care, Colombo, Sri Lanka; 4 Department of Psychological Medicine, Institute of Psychiatry, Psychology, and Neuroscience, King’s College London, London, UK; 5 NIHR Biomedical Research Centre for Mental Health at the South London and Maudsley NHS Foundation Trust, King’s College London, London, UK; 6 Department of Medicine, University of Rajarata, Mihintale, Sri Lanka; 7 Faculty of Medicine & Health Sciences, Research Institute for Primary Care & Health Sciences, Keele University, Newcastle-Under-Lyme, UK; 8 Psychology Department, Faculty of Social Sciences, Anton de Kom University of Suriname, Paramaribo, Suriname

**Keywords:** Nutrition, Food frequency, Cardio-metabolic risk indicators, Genetics, Twins, Sri Lanka

## Abstract

Low- and middle-income countries (LMICs) globally have undergone rapid urbanisation, and changes in demography and health behaviours. In Sri Lanka, cardio-vascular disease and diabetes are now leading causes of mortality. High prevalence of their risk factors, including hypertension, dysglycaemia and obesity have also been observed. Diet is a key modifiable risk factor for both cardio-vascular disease and diabetes as well as their risk factors. Although typically thought of as an environmental risk factor, dietary choice has been shown to be genetically influenced, and genes associated with this behaviour correlate with metabolic risk indicators. We used Structural Equation Model fitting to investigate the aetiology of dietary choices and cardio-metabolic phenotypes in COTASS, a population-based twin and singleton sample in Colombo, Sri Lanka. Participants completed a Food Frequency Questionnaire (N = 3934) which assessed frequency of intake of 14 food groups including meat, vegetables and dessert or sweet snacks. Anthropometric (N = 3675) and cardio-metabolic (N = 3477) phenotypes were also collected including weight, blood pressure, cholesterol, fasting plasma glucose and triglycerides. Frequency of consumption of most food items was found to be largely environmental in origin with both the shared and non-shared environmental influences indicated. Modest genetic influences were observed for some food groups (e.g. fruits and leafy greens). Cardio-metabolic phenotypes showed moderate genetic influences with some shared environmental influence for Body Mass Index, blood pressure and triglycerides. Overall, it seemed that shared environmental effects were more important for both dietary choices and cardio-metabolic phenotypes compared to populations in the Global North.

**Supplementary Information:**

The online version contains supplementary material available at 10.1007/s10519-023-10165-8.

## Introduction

In many Low- and middle-income countries (LMICs) rapid urbanization has resulted in changes in health behaviours (Yusuf et al. 2001a, b). In addition, the burden from non-communicable diseases (NCDs) and years lived with disability due to these diseases have risen (Popkin 2015; Richards et al. 2016). Globally, seventeen million deaths a year are attributable to NCDs and of all NCD deaths, 77% of these are in LMICs (Vos et al. 2020). For example, in Sri Lanka, cardio-vascular disease (CVD) is the leading cause of mortality and high prevalence is reported for its associated risk factors (Hills et al. 2018; Jayawardena et al. 2012; Ranasinghe et al. 2015, 2017). One potential modifiable risk factor for CVD and diabetes is diet. The relationship between nutritional choices and the risk factors for CVD and diabetes is reported in high as well as LMICs. It is, however, unclear whether this relationship is environmental in nature or due to a common genetic predisposition. Understanding the aetiology of individual differences in dietary choices, cardio-metabolic risk factors and their relationship is essential for informing intervention strategies.

### The aetiology of nutritional choices

Twin studies enable decomposition of the genetic and environmental influences on the variation of a trait. Whilst dietary choices are often thought to be environmental in origin, several twin studies have shown both genetic and environmental factors influencing individual differences in this trait. Twin studies investigating frequency of consumption of different food groups and related traits have typically been conducted in high income populations in the Global North. Genetic influences on self-reported frequency of consumption of 24 food items in a young adult Finnish sample ranged between 22 and 55%, with no significant effects of shared environmental influences (that is environmental factors that make family members more alike), and significant unique environmental influences (individual-specific environmental factors) (Keskitalo et al. 2008). Similar results were observed in a Dutch sample of older twins for a ‘healthy’ and ‘unhealthy’ dietary factor derived from 99 food items (van den Bree et al. 1999). Genetic influences of between 17 and 49% were reported for frequency of consumption of 24 food types in a sample of adult female twins (Teucher et al. 2007). However, a slightly different pattern of results were reported in a Danish study, where shared environmental effects were observed for the consumption of some food groups, such as vegetables, fruit, poultry and fish (39–44%) in males, and potatoes, vegetables, fruit, poultry, and candy/chocolate (23%–46%) in females (Hasselbalch et al. 2008).

Some studies have investigated nutritional intake rather than frequency of food consumption. In a US sample of adult twins reared apart genetic influences on nutrition/calorie intake ranged between 16 and 46% (Hur et al. 1998). A US study of early adolescent twins, which used a 3-day 24-h recall food diary to assess intake of total energy, macronutrients and micronutrients, reported genetic influences for dietary intake between 21 and 48%, with no effects of shared environmental factors (Liu et al. 2013). Two UK studies considered the more psychological aspects of diet, assessed by means of *preference* for different food groups and food/lifestyle patterns. They reported genetic influences between 32 and 58%, with no shared environmental effects (Pallister et al. 2015; Smith et al. 2016). In summary, research suggests some degree of genetic influence on food consumption frequency and nutritional intake, with little effect of shared environment but some indication of sex differences in aetiology. There are to date no twin studies on frequency of food consumption conducted in LMICs.

### The aetiology of cardio-metabolic risk factors

Our review of recent, well powered, twin studies found some evidence for the importance of shared environment on cardio-metabolic risk factors (CMR). In a young sample (8–17 years) from the Chinese Qingdao Twin registry (Ji et al. 2020), sex differences were observed for Body Mass Index (BMI), with higher heritability for males (63%) then females (38%). Shared environmental influences on BMI were only observed in females and were quite substantial (45%). For waist circumference and blood pressure, there was a high proportion of shared environmental effects in both men (63%) and women (52%). This is in contrast with studies conducted on BMI and CMR in Korea, and European and US studies, reporting high heritabilities and no effects of shared environment (Elder et al. 2009; Jermendy et al. 2011; Souren et al. 2007; Sung et al. 2009). However, comparison is hindered because these studies have generally focused on older populations and sex differences were not considered. In the Qingdao sample, the reported heritability for triglycerides was moderate (~ 40%) with substantial influence of shared environmental effects for males and females (~ 30%). For HDL cholesterol heritability was moderate to high (54–69%) with no effect of shared environment. In a sub-sample, the heritability estimate for fasting plasma insulin was reported to be 68% (Wang et al. 2020). These results are mostly in line with those from studies investigating similar phenotypes in Korea, Europe and the USA (Beekman et al. 2002; Goode et al. 2007; Poulsen et al. 2001; Souren et al. 2007; Sung et al. 2009; Wessel et al. 2007). In summary, it appears that the effects of shared environment are more pronounced in some Asian populations and, that there is some indication of sex differences in aetiology for BMI.

### Molecular genetic evidence for the link between dietary phenotypes and cardio-metabolic risk (CMR)

There are currently no twin studies that have directly investigated whether the same genetic factors influence both the consumption frequencies of food groups and cardio-metabolic risk factors. In most molecular genetic (nutrigenetic and nutrigenomic) studies, dietary phenotypes have been used as a mitigating ‘environmental’ factor of metabolic syndrome (and, thus, ignored the genetic nature of diet and a possible genetic link). One example is a study showing a significant interaction of the obesity risk gene MC4R rs17782313 with dietary quality indices on low-density lipoprotein cholesterol, LDL-C) in females and on serum glucose concentrations, systolic and diastolic blood pressure in males (Khodarahmi et al. 2020). In another, a significant interaction between a cholesterol gene (Caveoline -1) and dietary pattern on cardio-metabolic risk factors was reported (Abaj et al. 2021). Considering combined effects of multiple genes, a significant interaction effect between the genetic cardiometabolic risk score and a low-protein diet on BMI, waist circumference, and triglyceride levels among Southeast Asian women was reported (Alsulami et al. 2020). A comprehensive review of the evidence of 42 Genome Wide Association Studies (GWAS) reporting G × E interactions of lifestyle and dietary habits related to metabolic syndrome was recently published (San-Cristobal et al. 2022). Inclusion of the interaction terms in constructing genetic risk scores to increase their predictive power was proposed. In terms of molecular genetic evidence for a genetic correlation between dietary phenotypes and CMR: only one GWAS on prudent and western dietary patterns reported an association with genes previously associated with CVD risk factors and obesity (Guenard et al. 2017).

### The present study

The setting of our research is Sri Lanka, a LMIC with a multi-ethic population of 22 million people, 33.3% of whom are below 18 years of age. Sri Lanka has endured a three-decade civil war, and the Asian Tsunami in 2004, resulting in internally displaced people with increased rates of mental health conditions (Siriwardhana and Stewart 2013). Fourteen years post war, Sri Lanka is experiencing demographic transition, rapid globalization, urbanization, and internal/external migration. Sri Lanka, like many other LMICs, is also currently experiencing a nutritional transition, adopting some aspects of Westernized diet, with co-existence of both under-nutrition and obesity (Jayawardena et al. 2013). Diet in Sri Lanka has traditionally included high levels of vegetables and rice, with availability and affordability of different food items influencing their consumption. We set out to investigate the genetic and environmental basis of self-reported consumption frequency of certain food items in Sri Lanka and explored the phenotypic and aetiological associations between diet and cardio-metabolic risk factors, using a Sri Lankan population-based twin and singleton sample. Due to the lack of directly comparable research in this area, this study was exploratory in nature and had no pre-defined hypotheses regarding the etiology of the different food groups and their relationship with CMR.

## Methods

### Setting

#### Sri Lanka

Sri Lanka has a population of 22 million (World Bank, 2023). Sri Lanka is a multi-ethnic society where the majority (74%) is Sinhalese while (18%) are Tamil and (7%) Moor. The remaining 1% consists of Burgher, Malay and Veddas. Seventy percent of the population are Buddhists. The remaining are Hindus (15%), Christians (8%) and Muslims (7%). Vital statistics are amongst the best in South Asia, with a literacy rate of 96.9% in males and 94.6% in females, and life expectancy averaging 72 years in males and 78.6 years in females (DCS Sri Lanka, 2015). Crude death rate is 6.7per 1000 population and the maternal mortality rate is 25.7per 10,000 live births. Infant mortality is 8.5 per 1000 live births (MoH Sri Lanka, 2021). Twinning rate in Sri Lanka is 10 per 1000 live births.

#### Colombo District

The sample were recruited from the Colombo District in Sri Lanka. The Colombo district has a population of 2.32 million, composed of multiple ethnic groups, including Sinhalese (76.5%), Tamils (11.2%) and Moors (10.7%). It is mainly classified as urban (77.6%) and includes the capital city of Sri Lanka, Colombo, but the wider district also includes rural areas. Typical of many urban regions, the district attracts a high level of immigration, of which a substantial minority have been displaced as a result of conflict. The population is characterised by great socio-economic diversity in terms of education, employment, and occupational social class. Since data collection ended for the original COTASS study in 2007, the three decade-long civil conflict in Sri Lanka came to an end.

### Sample

Participants were recruited in the second wave of the Colombo Twin and Singleton Study (CoTaSS-2) study between 2012 and 2015. The CoTaSS-2 sample consisted of 73.9% (N = 2934) twins and 26.1% (N = 1035) singletons, 57.6% were female, and ages ranged from 19 to 91 years (mean age 42.8 years). Most of the sample were married and lived in urban areas. Of the twins, 461 pairs lived within the same household at the time of data collection. The dominant ethnic group was Sinhalese (92.7%), with Buddhism the most reported religion (86.5%). Over half of the sample were in employment, with the most frequently reported occupation being non-manual or skilled manual labour. Most CoTaSS-2 participants were educated beyond grade 6, and 5% reported university education. Data collection in CoTaSS-2 was accomplished via interviews, anthropometric measurements, and blood and urine sampling to conduct clinical investigations. Participant tracing, sample characteristics and data collection have been detailed elsewhere (Jayaweera et al. 2018).

### Measures & data collection

### Frequency of consumption of Food Groups Questionnaire (FFQ)

Questionnaire data were collected in interviews conducted by trained field research assistants at participants’ homes. Food consumption patterns were measured using a culturally adapted version of the FFQ, revised in consultation with local experts (Iqbal et al. 2008). Participants were asked to indicate the frequency of consumption of certain food groups per day as well as per week basing their responses on their behaviour in the previous week. We focus on weekly consumption as weekly consumption patterns are likely to be more informative than daily consumption which may vary depending on day of the week (e.g. weekend consumption vs weekday consumption). The food groups included were meat, fish, eggs, grains, rice, flour, dairy products, deep-fried foods, salty snacks, desserts/sweet snacks, nuts/seeds, fruits, leafy greens and vegetables. Examples of local Sri Lankan items were added for each food group in the questionnaire (see Table S1).

### Anthropometric & blood pressure measurement

Measurements were taken by trained field research assistants according to standard protocols, at the same time as the questionnaire data collection. Height and weight were measured to the closest 0.1 cm/Kg using portable stadiometers and weighing scales (Seca, Germany). BMI was calculated from standing height and weight. Waist Circumference was measured using measuring tapes. Blood pressure (systolic & diastolic) was measured using Omron HEM-7200 automatic blood pressure monitors (Omron Healthcare, Japan). Three blood pressure recordings were obtained after 10 min of rest with 2–3 min intervals between measurements. The mean across recordings was used in the analyses. Quality checks were done by random spot checking, and by the data entry team (Jayaweera et al. 2018).

### Cardio metabolic risk measurement

Fasting blood samples were collected during early morning home visits, at the research institute or other specified clinical laboratory. Blood samples were collected using evacuated blood collection tubes (Becton Dickinson, USA). Fasting blood glucose (mmol/l), fasting blood Insulin (µmol/ml), HDL cholesterol (mmol/l) and triglyceride levels (mmol/l) were extracted by a private clinical laboratory, using standard protocols (Jayaweera et al. 2018).

### Statistical analysis

Data cleaning, covariate-regression, transformations and generation of descriptive statistics were done in R (R Core Team 2021). For the FFQ, two outliers (eggs: 50 per week and deep-fried foods: 52 per week) were replaced by the mean (1.46 and 2.28, respectively). All items (apart from rice and dairy products) were log-transformed to reduce skew. For the CMR variables, replacing extreme values (i.e. > 3 SD from the mean) was considered, but due to a high number of outliers for triglycerides, fasting plasma glucose and insulin we instead report results without outliers. The CMR variables were log transformed to reduce skew.

Structural Equation Model-fitting (SEM) analysis were conducted in the R-based OpenMx package (Boker et al. 2011) to ascertain maximum likelihood estimates of the twin correlations and the latent genetic and environmental variance components implied by the genetically sensitive twin data (see Twin Design below). As is customary for twin analysis, the effects of sex and age were regressed out (McGue and Bouchard 1998) before transformation and models were fitted on raw data to facilitate missing data using Full Information Maximum Likelihood.

#### The twin design

The twin design uses the differential correlation in data collected from monozygotic (MZ) and dizygotic (DZ) twin pairs to estimate the extent to which the individual differences in a variable (variance) can be explained by genetic and environmental differences between people in a population. These underlying (latent) aetiological effects are based on a model where the MZ twin pairs are assumed to have a perfect correlation due to latent additive genetic effects (A), but the DZ pairs have a correlation of 0.5 due to sharing on average 50% of their segregating genes (like other siblings). In terms of the shared (family) environment (C), MZ and DZ pairs are correlated 100% if reared together. The environmental factor that is individual-specific (E) is uncorrelated in both MZ and DZ pairs and explains differences among individuals within the same family (as well as measurement error). The models for the expected correlations in MZ and DZ pairs (A + C and 0.5A + C, respectively) are fitted to the observed correlations in order to estimate the most likely estimates for A, C and E. This means that the ratio of the MZ and DZ correlations will indicate which model to expect. With a 2:1 ratio, only genetic influences are assumed, no effects of shared environment, whilst with a 1:1 ratio, only shared environmental effect are indicated, with no genetic influences. A combination of genetic and shared environmental effects is indicated for any ratio in between, i.e., if the DZ twin correlation is more than half that of the MZ twin pairs (McAdams et al. 2021; Rijsdijk and Sham 2002).

##### SEM procedure

To estimate the maximum-likelihood twin correlations using all (twin and singleton) data, we first fit a constrained correlation model in which the variances were specified for males and females separately across the twin and singleton data; and the variances were constrained across birth-order within same-sex pairs. Correlations were then estimated for the 5 sex-by-zygosity groups (MZ and DZ male pairs, MZ and DZ female pairs and DZ opposite-sex pairs).

Second, two full heterogeneity ACE models were fitted which estimate A, C and E separately for males and females (quantitative sex difference) as well as free correlations between either the A or C factors of males and females in opposite sex pairs (qualitative sex differences). Quantitative differences are indicated by differences in the MZ and DZ ratios in male and female twin pairs. Qualitative differences are indicated by significantly different opposite-sex vs same-sex DZ correlations. Third, a heterogeneity ACE model was fitted which fixed the correlations between the A and C factors of males and females of opposite sex pairs to 0.5 and 1, respectively (i.e., dropping the qualitative sex differences). Fourth, a homogeneity model in which A, C and E are equated across males and females was fitted and compared to previous models to infer sex differences in aetiology. To ensure that it is not a difference in the variance of the phenotypic trait that is accounting for potential sex-differences in A, C and E, the fit of a variance inequality (scalar) model was evaluated as well. Since the models are nested, their relative fit is established by the difference in minus twice the log likelihood (− 2LL) of the raw observations, which is distributed as chi-square. Confidence intervals for model parameters were obtained by maximum likelihood estimation.

## Results

Descriptive statistics for weekly consumption of food groups of the FFQ (raw untransformed data) are presented in Table 1. The effects of *demographic* and *socio-economic factors* on the consumption of different food groups are presented in the supplementary material (Table S2). Age significantly predicted food frequency for the majority of food groups. Where there was an effect, older age was associated with less consumption of these food groups. People living in semi-urban areas ate less deep-fried foods, fish, dairy and fruit, but more grains, rice, vegetables, sweet and salty snacks. Financial strain was associated with eating less meat, dairy, eggs, sweet snacks, greens, vegetables and fruit, but more fish. Being employed was associated with a higher consumption of meat, fruit and eggs, but also deep-fried foods and sweet snacks. Religion also affected dietary choices, possibly reflecting faith-based influences on typical meals consumed. Education had no observable impact on food choices.

**Table 1:** Descriptive statistics of the Frequency of Food Groups Questionnaire (FFQ) items (weekly consumption)

	N	Mean (SD)	Median	Range	Skew	Kurtosis
1. Meat	3927	1.73 (2.88)	1	0–24	3.10	13.15
2. Fish	3927	8.95 (6.28)	7	0–35	0.47	− 0.61
3. Eggs	3927	1.48 (2.09)	1	0–50	6.05	91.45
Eggs*	3927	1.46 (1.94)	1	0–21	3.57	23.81
4. Grains	3927	8.61 (5.99)	7	0–44	0.90	0.11
5. Rice	3926	18.03 (5.17)	21	0–38	− 0.86	0.91
6. Flour	3924	3.91 (4.25)	2	0–22	1.23	1.01
7. Dairy Products	3925	10.76 (7.02)	10	0–43	0.29	− 0.16
8. Deep-fried foods	3926	2.29 (3.83)	1	0–52	3.23	15.45
Deep-fried foods*	3926	2.28 (3.75)	1	0–21	2.87	9.30
9. Salty Snacks	3926	1.88 (3.64)	0	0–28	3.08	10.80
10. Dessert/sweet snacks	3926	5.46 (7.63)	2	0–51	1.74	3.11
11. Nuts/Seeds	3925	0.79 (1.58)	0	0–21	4.47	31.57
12. Fruits	3930	7.45 (5.93)	7	0–35	0.88	− 0.05
13. Leafy greens	3927	7.54 (5.27)	7	0–23	0.87	0.13
14. Vegetables	3925	16.91 (6.09)	15	0–60	1.02	− 0.53

*One outlier for Eggs (50 per week) and Deep-fried foods (52 per week) were replaced by the sample mean excluding the outliers. Variables are unadjusted and untransformed

Descriptive statistics for the CMR variables (raw and untransformed data) are presented in Table 2. For triglycerides, fasting plasma glucose and fasting plasma insulin results are also reported with outliers (3 SD above/below the mean) removed (N = 65, 91, 18, respectively).

**Table 2:** Descriptive statistics for Cardio Metabolic Risk variables

	N	Mean (SD)	Median	Range	Skew	Kurtosis
1. Waist circumference	3665	88.98 (11.58)	88.80	55.2–147.8	0.21	0.14
2. BMI	3659	23.74 (4.57)	23.51	12.61–49.76	0.49	0.44
3. Systolic blood pressure	3671	118.00 (18.81)	115	65–220	1.16	2.28
4. Diastolic blood pressure	3671	76.79 (11.08)	75.67	41.33–137.67	0.68	0.97
5. Triglycerides (mmol/l)	3472	1.44 (0.86)	1.22	0.33–13.41	3.25	22.92
Triglycerides (mmol/l)^a^	3407	1.37 (0.65)	1.21	0.33–3.99	1.26	1.45
6. HDL cholesterol (mmol/l)	3472	1.28 (0.25)	1.27	0.52–2.15	0.23	-0.27
7. Fasting plasma glucose (mmol/l)	3471	5.97 (2.31)	5.33	2.55–32.41	4.26	23.98
Fasting plasma glucose (mmol/l)^b^	3380	5.67 (1.34)	5.33	2.55–12.88	2.72	8.93
8. Fasting plasma insulin (µmol/ml)	3470	13.2 (18.84)	10	0.70–624.5	18.5	496.15
Fasting plasma insulin (µmol/ml)^c^	3452	12.36(9.22)	9.9	0.70–67.3	2.11	6.34

^a,b,c^Outliers (> 3 SD) removed, N = 65, 91, 18, respectively. Variables are unadjusted and untransformed

### Univariate twin analysis: FFQ

Maximum likelihood twin correlations for the FFQ items are given in Table 3. The MZ:DZ ratios in both males and females suggest that, in general, there are little heritable effects explaining individual differences in consumption of these food groups in Sri Lanka. The estimates of the best-fitting genetic models are presented in Table 4 (or Figure S1). For most of the items the predominant factor causing variation in consumption is the unique environment (48–86%). For some items there was also a significant contribution of shared environment and genetic factors. In general, no sex differences in aetiology were observed, apart from consumption of meat (more heritable in men, 27%; more effects of shared environment in females, 35%) and vegetables (the only source of familial effects in females are those due to shared environment, 50%; in males there is also some heritable effects, albeit non-significant). Variation in frequency of consumption of flour, fruit and leafy greens were mostly heritable (31%, 30% and 25%). Significant effects of shared (family) environment were observed for meat (females), fish, eggs, grains, rice, diary, deep fried foods, fruits and vegetables (14–50%). Shared environmental influences were highest for vegetables (50%), meat in females (35%), and grains (27%). Model-Fitting results and fit indices for the tested sex-differences model are detailed in the supplementary material (**Table S5**).

**Table 3:** Twin correlations for the frequency of food groups questionnaire items (weekly consumption)

	MZM	DZM	MZF	DZF	DZOS
Meat	**0.36 [0.26–0.46]**	**0.25 [0.10–0.38]**	**0.37 [0.28–0.45]**	**0.37 [0.25–0.48]**	**0.14 [0.05–0.22]**
Fish	**0.21 [0.09–0.32]**	**0.25 [0.10–0.38]**	**0.29 [0.18–0.38]**	**0.18 [0.02–0.32]**	**0.30 [0.20–0.40]**
Eggs	0.06 [− 0.05–0.17]	0.05 [− 0.12–0.20]	**0.16 [0.06–0.26]**	0.08 [− 0.04–0.19]	**0.28 [0.19–0.37]**
Grains	**0.37 [0.26–0.46]**	**0.19 [0.04–0.33]**	**0.38 [0.29–0.46]**	**0.33 [0.20–0.44]**	**0.39 [0.29–0.47]**
Rice	**0.49 [0.40–0.57]**	**0.30 [0.14–0.44]**	**0.30 [0.20–0.39]**	**0.29 [0.16–0.40]**	**0.35 [0.24–0.44]**
Flour	**0.39 [0.28–0.49]**	**0.29 [0.13–0.42]**	**0.39 [0.30–0.48]**	**0.22 [0.09–0.34]**	**0.23 [0.12–0.33]**
Dairy products	**0.26 [0.14–0.37]**	**0.36 [0.19–0.31]**	**0.44 [0.34–0.51]**	**0.19 [0.06–0.31]**	**0.29 [0.20–0.38]**
Deep fried foods	**0.24 [0.13–0.33]**	0.09 [− 0.09–0.25]	**0.34 [0.25–0.42]**	**0.26 [0.11–0.39]**	**0.26 [0.19–0.34]**
Salty snacks	**0.28 [0.17–0.37]**	0.13 [− 0.06–0.29]	**0.15 [0.04–0.26]**	0.15 [− 0.03–0.30]	**0.24 [0.13–0.34]**
Desserts/sweet snacks	**0.26 [0.14–0.36]**	**0.20 [0.05–0.34]**	**0.37 [0.27–0.46]**	0.15 [0.00–0.29]	**0.27 [0.18–0.36]**
Nuts/seeds	**0.21 [0.11–0.30]**	**0.30 [0.12–0.45]**	**0.15 [0.05–0.25]**	**0.27 [0.14–0.38]**	0.07 [− 0.01–0.17]
Fruits	**0.55 [0.46–0.63]**	**0.29 [0.14–0.42]**	**0.50 [0.42–0.57]**	**0.37 [0.24–0.47]**	**0.41 [0.33–0.49]**
Leafy greens	**0.39 [0.27–0.49]**	**0.20 [0.02–0.36]**	**0.36 [0.25–0.45]**	**0.33 [0.19–0.44]**	**0.22 [0.11–0.31]**
Vegetables	**0.35 [0.23–0.45]**	**0.51 [0.35–0.62]**	**0.43 [0.34–0.51]**	**0.66 [0.57–0.72]**	**0.30 [0.20–0.40]**

Estimates based on a constrained correlation model (equal means and variances across birth-order and zygosity group, within sex) fitted on age and sex adjusted variables

The sample size for the FFQ data was 508 MZM, 348 DZM, 709 MZF, 476 DZF individuals; 378 DZOS males, 409 DZOS females

Significant correlations are shown in bold

**Table 4:** Best-fitting univariate ACE model estimates for Frequency of Food Groups Questionnaire items (weekly consumption)

	A	C	E
Meat (Quantitative Heterogeneity ACE)			
Male	**0.27 [0.05–0.45]**	0.09 [0.00–0.29]	**0.63 [0.54–0.73]**
Female	0.03 [0.00–0.25]	**0.35 [0.14–0.43]**	**0.63 [0.55–0.70]**
Fish (Homogeneity ACE)	0.00 [0.00–0.20]	**0.25 [0.10–0.31]**	**0.75 [0.67–0.80]**
Eggs (No-sex-dif Variance inequality ACE)	0.00 [0.00–0.12]	**0.14 [0.04–0.19]**	**0.86 [0.80–0.91]**
Grains (No-sex-dif Variance inequality ACE)	0.11 [0.00–0.29]	**0.27 [0.12–0.39]**	**0.62 [0.56–0.69]**
Rice (No-sex-dif Variance inequality ACE)	0.14 [0.00–0.32]	**0.25 [0.09–0.38]**	**0.61 [0.55–0.68]**
Flour (Homogeneity ACE)	**0.31 [0.12–0.46]**	0.08 [0.00–0.23]	**0.61 [0.54–0.67]**
Dairy products (No-sex-dif Variance Inequality ACE)	0.17 [0.00–0.37]	**0.19 [0.03–0.33]**	**0.64 [0.57–0.71]**
Deep fried foods (No-sex-dif Variance Inequality ACE)	0.11 [0.00–0.29]	**0.18 [0.04–0.30]**	**0.71 [0.64–0.78]**
Salty snacks (No-sex-dif Variance Inequality ACE)	0.04 [0.00–0.26]	0.18 [0.00–0.26]	**0.79 [0.71–0.85]**
Desserts/sweet snacks (No-sex-dif Variance Inequality ACE)	0.18 [0.00–0.37]	0.14 [0.00–0.29]	**0.68 [0.61–0.76]**
Nuts/seeds (No-sex-dif Variance Inequality ACE)	0.04 [0.00–0.23]	0.14 [0.00–0.22]	**0.82 [0.75–0.88]**
Fruits (Homogeneity ACE)	**0.30 [0.13–0.46]**	**0.23 [0.09–0.36]**	**0.48 [0.42–0.54]**
Leafy greens (Homogeneity ACE)	**0.25 [0.05–0.43]**	0.12 [0.00–0.28]	**0.63 [0.56–0.71]**
Vegetables (Quantitative Heterogeneity ACE)			
Male	0.13 [0.00–0.31]	**0.25 [0.12–0.39]**	**0.62 [0.53–0.72]**
Female	0.00 [0.00–0.11]	**0.50 [0.38–0.56]**	**0.50 [0.44–0.57]**

Quantitative Heterogeneity model: the ratios of the MZ and DZ correlations in males and females suggest different ACE contributions (amounts) to the observed variance; Homogeneity model: the ratios of the MZ and DZ correlations in males and females suggest the same ACE contributions (amounts) to the observed variance; No-sex-dif Variance Inequality model: the ratios of the MZ and DZ correlations in males and females suggest the same ACE contributions (amounts) to the observed variance, but the variances are significantly different. If a scalar is introduced to adjust for this variance inequality, there are no sex differences in aetiology. The sample size for the FFQ data was 508 MZM, 348 DZM, 709 MZF, 476 DZF individuals; 378 DZos males, 409 DZos females; and 388 and 639 non-twin ‘singleton’ males and females, respectively

Significant effects are shown in bold

### Univariate twin analysis: Cardio Metabolic Risk Variables

Maximum likelihood twin correlations for the CMR variables are given in Table 5. The estimates of the best-fitting genetic model are presented in Table 6 (or **Figure S2**). The MZ:DZ ratios in both males and females suggest that there is a fair proportion of heritable effects on the variance of CMR variables (28–64%), apart from triglycerides and fasting plasma Insulin in males, which suggests higher effects of the shared environment (31% and 34%). There are quantitative sex differences in aetiology for most variables, apart from HDL cholesterol and fasting plasma glucose, which also are the most heritable (55% and 64%, respectively). The most familial variables (sum of A and C effects) are waist circumference and BMI (~ 80%). Model-Fitting results and fit indices for the tested sex-differences model are detailed in the supplementary material (**Table S6**).

**Table 5:** Twin correlations for cardio metabolic risk variables

	MZM	DZM	MZF	DZF	DZOS
Waist circumference (cm)	**0.76 [0.71–0.81]**	**0.56 [0.42–0.66]**	**0.72 [0.67–0.77]**	**0.50 [0.40–0.59]**	**0.24 [0.14–0.34]**
BMI	**0.77 [0.72–0.81]**	**0.55 [0.42–0.65]**	**0.82 [0.78–0.85]**	**0.55 [0.44–0.63]**	**0.30 [0.20–0.40]**
BP Systolic	**0.52 [0.41–0.60]**	**0.25 [0.08–0.40]**	**0.64 [0.57–0.70]**	**0.34 [0.21–0.45]**	0.07 [− 0.05–0.18]
BP Diastolic	**0.51 [0.40–0.60]**	**0.30 [0.14–0.44]**	**0.59 [0.51–0.65]**	**0.29 [0.15–0.40]**	0.02 [− 0.10–0.13]
Triglycerides (mmol/l)	**0.55 [0.49–0.63]**	**0.37 [0.18–0.51]**	**0.57 [0.50–0.64]**	**0.32 [0.16–0.45]**	**0.21 [0.09–0.32]**
Triglycerides (mmol/l)^a^	**0.44 [0.31–0.55]**	**0.37 [0.19–0.51]**	**0.58 [0.51–0.65]**	**0.26 [0.10–0.40]**	0.09 [− 0.03–0.20]
HDL cholesterol (mmol/l)	**0.57 [0.46–0.65]**	**0.31 [0.15–0.45]**	**0.57 [0.49–0.63]**	**0.28 [0.13–0.41]**	**0.15 [0.03–0.27]**
Fasting plasma glucose (mmol/l)	**0.71 [0.63–0.77]**	0.27 [− 0.07–0.48]	**0.64 [0.57–0.70]**	0.13 [− 0.04–0.29]	**0.22 [0.08–0.34]**
Fasting plasma glucose (mmol/l)^b^	**0.60 [0.49–0.68]**	0.14 [− 0.09–0.34]	**0.68 [0.60–0.73]**	0.14 [− 0.05–0.31]	**0.36 [0.25–0.46]**
Fasting plasma Insulin (µmol/ml)	**0.48 [0.38–0.57]**	**0.41 [0.26–0.54]**	**0.52 [0.43–0.60]**	**0.26 [0.12–0.39]**	**0.17 [0.05–0.28]**
Fasting plasma Insulin (µmol/ml)^c^	**0.44 [0.34–0.54]**	**0.49 [0.33–0.60]**	**0.49 [0.39–0.57]**	**0.24 [0.11–0.36]**	**0.19 [0.07–0.30]**

Estimates of a constrained correlation model (equal means and variances across birth-order and zygosity group, within sex) estimated on age and sex adjusted variables

^a,b,c^Outliers (> 3 SD) removed, N = 65, 91, 18, respectively

The sample size for the anthropometric and cardio-metabolic data was 483 MZM, 328 DZM, 651 MZF, 441 DZF individuals; 361 DZOS males, 385 DZOS females

Significant correlations are shown in bold

**Table 6:** Best-fitting univariate ACE model estimates for Cardiometabolic Risk Variables

	A	C	E
Waist circumference (cm) (Quantitative Heterogeneity ACE, with Qualitative A or C effects*)			
Male	**0.41 [0.19–0.70]**	**0.35 [0.07–0.56]**	**0.24 [0.19–0.29]**
Female * rA_MF_ = − .18 (NA/.20); rC_MF_ = − .09 (− .41/.56)	**0.44 [0.24–0.66]**	**0.29 [0.07–0.46]**	**0.28 [0.23–0.33]**
BMI (Quantitative Heterogeneity ACE, with Qualitative A or C effects*)			
Male	**0.45 [0.23–0.72]**	**0.32 [0.06–0.52]**	**0.23 [0.19–0.28]**
Female * rA_MF_ = − .19 (− .51/.30); rC_MF_ = − .01 (− .45/.30)	**0.54 [0.36–0.75]**	**0.28 [0.07–0.44]**	**0.18 [0.15–0.22]**
BP Systolic (Quantitative Heterogeneity ACE)			
Male	**0.37 [0.11–0.53]**	**0.13 [0.01–0.34]**	**0.50 [0.41–0.61]**
Female	**0.51 [0.30–0.66]**	0.12 [0.00–0.30]	**0.37 [0.31–0.44**]
BP Diastolic (Quantitative Heterogeneity ACE)			
Male	**0.28 [0.01–0.48]**	**0.21 [0.05–0.43]**	**0.51 [0.42–0.62]**
Female	**0.48 [0.28–0.62]**	0.10 [0.00–0.27]	**0.42 [0.35–0.50]**
Triglycerides (mmol/l) (No-sex-dif Variance inequality ACE)			
Male & Female	**0.56 [0.40–0.61]**	0.00 [0.00–0.14]	**0.44 [0.39–0.49]**
Scalar in females = 0.67			
Triglycerides (mmol/l)^a^ (Quantitative Heterogeneity ACE)			
Male	0.13 [0.00–0.43]	**0.31 [0.05–0.49]**	**0.56 [0.46–0.68]**
Female	**0.57 [0.39–0.64]**	0.01 [0.00–0.17]	**0.42 [0.36–0.50]**
HDL cholesterol (mmol/l) (Homogeneity ACE)	**0.55 [0.45–0.61]**	0.00 [0.00–0.08]	**0.45 [0.39–0.50]**
Fasting plasma glucose (mmol/l)			
(Homogeneity ACE)	**0.64 [0.58–0.69]**	0.00 [0.00–0.05]	**0.36 [0.31–0.42]**
Fasting plasma glucose (mmol/l)^b^ (Homogeneity ACE)	**0.64 [0.53–0.69]**	0.00 [0.00–0.09]	**0.36 [0.31–0.42]**
Fasting plasma Insulin (µmol /ml) (Quantitative Heterogeneity ACE)			
Male	0.14 [0.00–0.48]	**0.34 [0.04–0.52]**	**0.52 [0.43–0.62]**
Female	**0.52 [0.21–0.60]**	0.00 [0.00–0.10]	**0.48 [0.40–0.57]**
Fasting plasma Insulin (µmol /ml)^c^ (Quantitative Heterogeneity ACE)			
Male	0.01 [0.00–0.29]	**0.45 [0.19–0.54]**	**0.54 [0.46–0.63]**
Female	**0.43 [0.22–0.55]**	0.05 [0.00–0.23]	**0.52 [0.44–0.61]**

^a,b,c^Outliers (> 3 SD) removed, N = 65, 91, 18, respectively. Quantitative Heterogeneity model: the ratios of the MZ and DZ correlations in males and females suggest different ACE contributions (amounts) to the observed variance; Homogeneity model: the ratios of the MZ and DZ correlations in males and females suggest the same ACE contributions (amounts) to the observed variance; No-sex-dif Variance Inequality model: the ratios of the MZ and DZ correlations in males and females suggest the same ACE contributions (amounts) to the observed variance, but the variances are significantly different. If a scalar is introduced to adjust for this variance inequality, there are no sex differences in aetiology. Quantitative Heterogeneity with Qualitative A or C effects model: the DZ opposite sex correlations are significantly different from the DZ same sex correlations, indicating qualitative different effects across males and females. In addition, the ratios of the MZ and DZ correlations in males and females could suggest different ACE contributions (amounts) to the observed variance. The sample size for the anthropometric and cardio-metabolic data was 483 MZM, 328 DZM, 651 MZF, 441 DZF individuals; 361 DZOS males, 385 DZOS females; and 353 and 566 non-twin ‘singleton’ males and females, respectively

Significant effects are shown in bold

### Univariate twin analysis: height and weight

Maximum likelihood twin correlations and estimates of the best-fitting genetic model are presented in **Tables S3** and **S4** (or Figure S1), respectively. Interestingly, there is a significant amount of shared-environmental variance in females (42% and 35%) in addition to a significant proportion of heritable variance (48%) for height and weight. In males the familial effects for height and weight were only due to differences in genetic factors (88% and 78%, respectively), which is more comparable to results in western populations.

### Phenotypic correlations: frequency of food group consumption and Cardio Metabolic Risk Variables

Pearson correlations between FFQ items and CMR variables are reported in **Table S7**. Only 4 significant (but very low) correlations (in unexpected direction) were observed. These were between fasting glucose levels and rice, dairy, and sweet snacks (− 0.04, − 0.05, − 0.05, respectively); between diastolic blood pressure and dairy products (− 0.04), fruit consumption and waist circumference and BMI (0.06 and 0.04, respectively) and fish consumption and waist circumference (0.04). Given these small effect sizes, no further bivariate genetic model-fitting analyses were conducted.

## Discussion

The aim of this paper was to investigate the aetiology of frequency of consumption of certain food groups and CMR variables in the Sri Lankan population, and to investigate the phenotypic and aetiological link between them. Genetic influences were evident for the consumption frequency of flour, fruits, leafy greens and meat in males. The consumption frequency of salty and sweet snacks, as well as nuts/seeds, did not seem to be familial, and was mainly due to individual-specific environmental factors, including measurement error (68–82%). Our results seem to be in contrast with those reported in the Global North where more significant genetic effects are found for consumption of most food items (Keskitalo et al. 2008, Teucher et al. 2007, van den Bree et al. 1999). Instead, we found an increased impact of the environment (shared and non-shared) in determining individual differences in frequency of food consumption in Sri Lanka.

In Sri Lanka, and similar to other countries in South Asia, the typical diet consists of dairy, fish, meat and vegetables (Mihiranie et al. 2020). This is reflected in the results of the current sample which show rice, fish, dairy and vegetable/fruit to be the most commonly consumed food group. There is an environmental influence on availability of fish and meats in that people living in coastal areas of the country will generally consume more fish compared with more central parts of the Colombo district. Prior to the recent economic crisis meat, fish, vegetables and fruits were generally available and affordable. Whilst religious practices preclude the consumption of certain meat (e.g. Buddists and Hindus will tend to avoid beef), they do not restrict meat consumption overall. Vegetarianism and veganism is uncommon in Sri Lanka, we did however, see relatively low levels of meat consumption in our sample.

Overall, we found that environmental influences, both shared and non-shared, explained a large proportion of variance in frequency of consumption of food groups. Our finding that shared environment explained a significant proportion of variance (small to moderate) for the majority of food groups was notable, and could be explained by the greater family social support system in Sri Lanka and the higher rates of multigenerational households. Children in Sri Lanka are also likely to live within the family home for longer, often moving out only after marriage. It is therefore possible that this increased connectivity between family members explains some of the high shared environment that we find in our analyses. Non-shared environment was also important in explaining variance in consumption. This suggest that other environmental factors were important, however, it is also possible that the large non-shared environmental component is explained by measurement error. There is no clear reason why certain food groups (e.g. frequency of meat consumption and fruit consumption) display higher heritability than others. Although the effects could be mediated by genetic effects on taste perception (Matison et al., 2023). Further qualitative and quantitative work in Sri Lanka is needed to fully understand this finding.

In accordance with a Chinese twin study (Ji et al. 2020), for waist circumference and BMI we found significant effects of shared environment. This is in contrast with studies in Korea and other studies in the Global North (Beekman et al. 2002; Goode et al. 2007; Sung et al. 2009) where there is little or no effect of shared environment. The effect of shared environment was less for Blood pressure and zero for Triglycerides, HDL cholesterol and fasting plasma insulin, which had comparable heritabilities with other studies. Thus, the most noteworthy results are the significant effects of shared environment for waist circumference, BMI (~ 30%) in addition to significant heritable effects (~ 40%). The increased impact of shared environment in determining individual differences in BMI and waist circumference could be related to the same factors that determine frequency of food consumption in Sri Lanka. These may include the strong family support system which may mean that more meals are still shared as adults which influences both food consumption and BMI and weight circumference.

We did not find strong phenotypic associations between frequency of consumption of food items and CMR factors in our study, therefore no bivariate genetic analyses were conducted to investigate the aetiological link. This could be associated with the nature of the food frequency questionnaire which focus on eating behaviours in the previous week. Individuals who have cardio-metabolic risk factors may have, due to their health condition, changed their diet to reduce risk of cardio-vascular outcomes. It is also possible that due to the relatively young age of the sample and more recent changes in diet in Sri Lanka, these associations are not yet apparent.

### Strengths and limitations

The results need to be viewed considering several limitations. First, there is concern surrounding the validity of food frequency questionnaires to assess diet (Byers 2001). However, FFQs are commonly used in epidemiological studies and whilst we recognize the limitations of this type of dietary assessment, it is an effective way of establishing dietary patterns in a large sample of individuals. Second, the self-reported frequency of food consumption may have been affected by memory recall or social-desirability bias. Third, our assessment of frequency of food consumption is likely to only represent the previous week, and this may not be representative of general consumption patterns. The cross-sectional nature of the study does not allow us to determine whether participants changed their diet because of health reasons or identification of elevated cardio-vascular risk factors. Generalizability of results needs to be considered; while the sample is representative of people living in the Colombo district of Sri Lanka, it may not be representative of different regions of Sri Lanka. The inclusion of a singleton cohort is a strength of this study because whilst twins are generally representative of the general population we do note a higher prevalence of cardiometabolic indicators in singletons compared to twins in the current sample (Jayaweera et al. 2018).

## Conclusions

This twin study offers a unique insight into the aetiology of diet and CMR in a LMIC. The largest meta-analysis of twin studies confirmed that there is an over-representation of twin research in developed countries (Polderman et al. 2015). Twin studies in LMICs are essential for understanding the development of health conditions in countries with different cultural norms and different levels of social inequality and socio-economic deprivation, as these can affect the aetiology of human behaviours and diseases. This study suggests that in Sri Lanka environmental factors, rather than genetic factors, explain the majority of variance in food choices. The results should be considered as time-specific. A recent economic crash in Sri Lanka (2021) has had an ongoing effect on the availability and affordability of food which has affected food consumption. More research in this area is needed to fully understand the differences in etiology across the different food groups and its relationship with CMR.

### Supplementary Information

Below is the link to the electronic supplementary material.

Supplementary file1 (DOCX 182 KB)
