# Supplementary material for: Frequency of Consumption of Food Groups and Cardio-Metabolic Risk Factors: A Genetically Informative Twin Study in Sri Lanka
Source: Behav Genet. 2023 Dec 22;54(1):73–85. doi: 10.1007/s10519-023-10165-8 (PMC10822787; doi:10.1007/s10519-023-10165-8)
Supplement: Supplementary file 1 — Supplementary file1 (DOCX 182 KB) [file 10519_2023_10165_MOESM1_ESM.docx]

**Table S1 Frequency of Consumption of Food Groups Questionnaire (FFQ) adapted to include examples of Sri Lankan foods**

| **Food items** | **Daily** | **Weekly** |
| --- | --- | --- |
| 1. **Meat** :- Chicken, Beef, Pork, Mutton |  |  |
| 1. **Fish** :- Prawns, Crabs, Small fish, Large fish, Dried fish, tinned fish |  |  |
| 1. **Eggs** :- Chicken eggs, Quail eggs |  |  |
| **4. Grains**:- Gram, Green gram, Dhal, Kauppi, Dhal, Barley, Sago, Corn, Kurukkan, Sesame, Millet, Soya |  |  |
| **5. Rice**:- Rice, Food made from rice flour |  |  |
| **6. Flour**:- Bread and other food made using flour |  |  |
| **7. Dairy products**:- Milk (powdered or liquid), Ice cream, Yogurt, Curd, Cheese, Butter |  |  |
| **8. Deep-fried foods**:- Rolls, Patties, Pastries, Cutlets, Wade Chips, Fish, Meat, Potatoes , Murukku, Dried fish |  |  |
| **9. Salty snacks**: - Potato chips, Fried food, Different types of Murukku, Salty biscuits, Fried cassava (manioc) |  |  |
| **10. Desserts/Sweet snacks**: - Jelly, Pudding, Watalappan, Chocolate, Cake, caramel pudding, Sugar, Different types of syrup, Dhosi, Toffee, Dodol, Bhundi, Marshmallow |  |  |
| **11. Nuts/seeds**:- Peanuts, Cashew nuts |  |  |
| **12. Fruits**:- Pineapple, Apple, Orange, Grapes, Dragon fruit, Banana, Avocado, Mango, Guava, Mandarin, Wood apple, Papaya, Water melon, Pomegranate, Veralu, Plums, Waraka, Beli fruit, Orange juice, Mango juice, Wood apple juice. |  |  |
| **13. Leafy greens**:- Gotukola, Kathurumurunga, Salad leaves, Kankun, Mukunuwenna, Thampala, spinach. |  |  |
| **14. Vegetables**:- Tomato, Onion, Bell peppers, Garlic, Beet root, Carrot, Cabbage, Beans, Cucumber, Leeks, Brinjal, Ladies fingers, Bitter gourd, Pumpkin, Snake gourd, Ridge gourd (watakolu, Drum stick (murunga), Banana Peppers, Long bean, Thalana batu, Jack fruit, Ash plantain, Mushrooms, Cauliflower, Kakiri, Mango, Amberalla, Banana flower, Potato, Sweet potato. |  |  |

**Note:** Participants were asked how much of each of the item they had in the past week on a weekly basis and also on a daily basis during that week

**Table S2. Regression between food groups and age, sex, urbanicity, financial strain, education level, and employment status**

| **Meat** | ***N*** | ***β*** | ***p-value*** |
| --- | --- | --- | --- |
|  |  |  |  |
| Age (linear) | 3933 | -0.03 | **<.001** |
| Age (quartiles) base= 19-31 years | 1028 |  |  |
| 32-41 years | 1017 | -.56 | **<.001** |
| 42-53 years | 964 | -.66 | **<.001** |
| 54-91 years | 925 | -1.29 | **<.001** |
| Sex, base = Male | 1681 |  |  |
| Female | 2288 | -0.99 | **<.001** |
| Region, base = Urban | 2390 |  |  |
| Rural | 532 | -0.07 | .604 |
| Mixed | 826 | -0.38 | .**001** |
| Outside Colombo | 221 | -0.25 | .160 |
| Financial strain, base = Living comfortably | 3933 |  |  |
| Doing alright | 365 | -0.33 | .065 |
| Just about getting by | 2616 | -0.79 | **<.001** |
| Difficult to make ends meet | 284 | -0.53 | .021 |
| V difficult to make ends meet | 129 | -1.19 | **<.001** |
| Education, base = No education | 47 |  |  |
| Grade 1-5 | 274 | -0.23 | .424 |
| Grade 6-O/Ls | 1757 | 0.23 | .382 |
| Passed O/Ls | 632 | 0.52 | .074 |
| Up to/Passed A/Ls | 929 | 0.80 | .005 |
| University or higher | 276 | 0.78 | .023 |
| Other | 15 | 0.75 | .294 |
| Religion, base=Buddist | 3401 |  |  |
| Hindu | 64 | -.40 | .023 |
| Islam | 159 | 1.74 | **<.001** |
| Christian | 309 | .96 | **<.001** |
| Employment, base = Not in employment (incl. students) | 1758 |  |  |
| Employed | 2207 | 0.86 | **<.001** |
|  |  |  |  |

| **Fish** |  |  |  |
| --- | --- | --- | --- |
| Age (linear) | 3933 | -0.003 | .702 |
| Age (quartiles) base= 19-31 years | 1028 |  |  |
| 32-41 years | 1017 | .603 | 0.053 |
| 42-53 years | 964 | .918 | **0.003** |
| 54-91 years | 925 | -.047 | 0.879 |
| Sex, base = Male | 1681 |  |  |
| Female | 2288 | -0.05 | .797 |
| Region, base = Urban | 2390 |  |  |
| Rural | 532 | 0.48 | .125 |
| Mixed | 826 | -1.54 | **<.001** |
| Outside Colombo | 221 | -0.46 | .297 |
| Financial strain, base = Living comfortably | 3933 |  |  |
| Doing alright | 365 | 1.23 | **<.001** |
| Just about getting by | 2616 | 0.67 | .081 |
| Difficult to make ends meet | 284 | 0.19 | .659 |
| V difficult to make ends meet | 129 | 0.06 | .928 |
| Education, base = No education | 47 |  |  |
| Grade 1-5 | 274 | 0.98 | .287 |
| Grade 6-O/Ls | 1757 | 1.78 | .035 |
| Passed O/Ls | 632 | 0.16 | .855 |
| Up to/Passed A/Ls | 929 | 1.43 | .097 |
| University or higher | 276 | 1.10 | .249 |
| Other | 15 | 0.56 | .754 |
| Religion, base=Buddism | 3401 |  |  |
| Hindu | 64 | -2.99 | **<.001** |
| Islam | 159 | -1.81 | **<.001** |
| Christian | 309 | -0.09 | 0.82 |
| Employment, base = Not in employment (incl. students) | 1758 |  |  |
| Employed | 2207 | 0.52 | .013 |
|  |  |  |  |

| **Eggs** |  |
| --- | --- |

| Age (Linear) | 3933 | -0.22 | **<.001** |
| --- | --- | --- | --- |
| Age (quartiles) base= 19-31 years | 1028 |  |  |
| 32-41 years | 1017 | -.13 | 0.21 |
| 42-53 years | 964 | -.44 | **<.001** |
| 54-91 years | 925 | -.83 | **<.001** |
| Sex, base = Male | 1681 |  |  |
| Female | 2288 | -0.31 | **<.001** |
| Region, base = Urban | 2390 |  |  |
| Rural | 532 | -0.18 | .018 |
| Mixed | 826 | -0.14 | .069 |
| Outside Colombo | 221 | -0.07 | .559 |
| Financial strain, base = Living comfortably | 3933 |  |  |
| Doing alright | 365 | -0.24 | .025 |
| Just about getting by | 2616 | -0.36 | .005 |
| Difficult to make ends meet | 284 | -0.31 | .024 |
| V difficult to make ends meet | 129 | -0.70 | **<.001** |
| Education, base = No education | 47 |  |  |
| Grade 1-5 | 274 | 0.10 | .624 |
| Grade 6-O/Ls | 1757 | 0.25 | .121 |
| Passed O/Ls | 632 | 0.47 | .005 |
| Up to/Passed A/Ls | 929 | 0.50 | **.003** |
| University or higher | 276 | 0.73 | **.009** |
| Other | 15 | 0.43 | .220 |
| Religion, base=Buddism | 3401 |  |  |
| Hindu | 64 | 0.14 | .428 |
| Islam | 159 | 0.08 | .536 |
| Christian | 309 | 0.43 | **.001** |
| Employment, base = Not in employment (incl. students) | 1758 |  |  |
| Employed | 2207 | 0.41 | **<.001** |
|  |  |  |  |

| **Grains** |  |
| --- | --- |

| Age (Linear) | 3933 | 0.02 | .021 |
| --- | --- | --- | --- |
| Age (quartiles) base= 19-31 years | 1028 |  |  |
| 32-41 years | 1017 | -.48 | 0.11 |
| 42-53 years | 964 | .27 | 0.38 |
| 54-91 years | 925 | .43 | 0.16 |
| Sex, base = Male | 1681 |  |  |
| Female | 2288 | -0.46 | .026 |
| Region, base = Urban | 2390 |  |  |
| Rural | 532 | 0.15 | .587 |
| Mixed | 826 | -1.60 | **<.001** |
| Outside Colombo | 221 | -0.88 | .022 |
| Financial strain, base = Living comfortably | 3933 |  |  |
| Doing alright | 365 | 1.68 | **<.001** |
| Just about getting by | 2616 | 0.37 | .305 |
| Difficult to make ends meet | 284 | 0.04 | .928 |
| V difficult to make ends meet | 129 | -1.43 | .005 |
| Education, base = No education | 47 |  |  |
| Grade 1-5 | 274 | 0.41 | .689 |
| Grade 6-O/Ls | 1757 | 0.51 | .600 |
| Passed O/Ls | 632 | -0.50 | .611 |
| Up to/Passed A/Ls | 929 | 1.02 | .297 |
| University or higher | 276 | 1.39 | .186 |
| Other | 15 | 0.38 | .832 |
| Religion, base=Buddism | 3401 |  |  |
| Hindu | 64 | -1.97 | **0.012** |
| Islam | 159 | -2.87 | **<.001** |
| Christian | 309 | -1.29 | **<.001** |
| Employment, base = Not in employment (incl. students) | 1758 |  |  |
| Employed | 2207 | 0.25 | .224 |
|  |  |  |  |

| **Rice** |  |
| --- | --- |

| Age (Linear) | 3933 | -0.004 | .576 |
| --- | --- | --- | --- |
| Age (quartiles) base= 19-31 years | 1028 |  |  |
| 32-41 years | 1017 | .25 | .320 |
| 42-53 years | 964 | .12 | .630 |
| 54-91 years | 925 | -.07 | .773 |
| Sex, base = Male | 1681 |  |  |
| Female | 2288 | 0.02 | .908 |
| Region, base = Urban | 2390 |  |  |
| Rural | 532 | 3.99 | **<.001** |
| Mixed | 826 | 1.86 | **<.001** |
| Outside Colombo | 221 | 1.55 | **<.001** |
| Financial strain, base = Living comfortably | 3933 |  |  |
| Doing alright | 365 | 0.86 | .004 |
| Just about getting by | 2616 | -0.66 | .072 |
| Difficult to make ends meet | 284 | -0.37 | .355 |
| V difficult to make ends meet | 129 | -1.55 | .008 |
| Education, base = No education | 47 |  |  |
| Grade 1-5 | 274 | -0.26 | .798 |
| Grade 6-O/Ls | 1757 | 0.66 | .482 |
| Passed O/Ls | 632 | -0.16 | .870 |
| Up to/Passed A/Ls | 929 | 0.71 | .451 |
| University or higher | 276 | 0.25 | .803 |
| Other | 15 | 1.54 | .234 |
| Religion, base=Buddism | 3401 |  |  |
| Hindu | 64 | -4.15 | **<.001** |
| Islam | 159 | -5.64 | **<.001** |
| Christian | 309 | -2.51 | **<.001** |
| Employment, base = Not in employment (incl. students) | 1758 |  |  |
| Employed | 2207 | 0.22 | .199 |
|  |  |  |  |

| **Flour** |  |
| --- | --- |

| Age | 3933 | -0.01 | .005 |
| --- | --- | --- | --- |
| Age (quartiles) base= 19-31 years | 1028 |  |  |
| 32-41 years | 1017 | -.76 | **<.001** |
| 42-53 years | 964 | -1.02 | **<.001** |
| 54-91 years | 925 | -.63 | **<.001** |
| Sex, base = Male | 1681 |  |  |
| Female | 2288 | -0.23 | .112 |
| Region, base = Urban | 2390 |  |  |
| Rural | 532 | -0.54 | .007 |
| Mixed | 826 | -1.56 | **<.001** |
| Outside Colombo | 221 | -1.80 | **<.001** |
| Financial strain, base = Living comfortably | 3933 |  |  |
| Doing alright | 365 | -0.27 | .274 |
| Just about getting by | 2616 | -0.19 | .514 |
| Difficult to make ends meet | 284 | -0.81 | .014 |
| V difficult to make ends meet | 129 | -0.11 | .806 |
| Education, base = No education | 47 |  |  |
| Grade 1-5 | 274 | -0.71 | .335 |
| Grade 6-O/Ls | 1757 | -0.66 | .344 |
| Passed O/Ls | 632 | -0.75 | .293 |
| Up to/Passed A/Ls | 929 | -0.82 | .245 |
| University or higher | 276 | -0.45 | .543 |
| Other | 15 | -1.46 | .147 |
| Religion, base=Buddism | 3401 |  |  |
| Hindu | 64 | 3.11 | **<.001** |
| Islam | 159 | 5.04 | **<.001** |
| Christian | 309 | 1.57 | **<.001** |
| Employment, base = Not in employment (incl. students) | 1758 |  |  |
| Employed | 2207 | 0.13 | .355 |
|  |  |  |  |

| **Dairy Products** |  |
| --- | --- |

| Age (linear) | 3933 | -0.02 | .003 |
| --- | --- | --- | --- |
| Age (quartiles) base= 19-31 years | 1028 |  |  |
| 32-41 years | 1017 | -.75 | 0.032 |
| 42-53 years | 964 | -1.81 | **<.001** |
| 54-91 years | 925 | -1.25 | **<.001** |
| Sex, base = Male | 1681 |  |  |
| Female | 2288 | -0.07 | .767 |
| Region, base = Urban | 2390 |  |  |
| Rural | 532 | -2.21 | **<.001** |
| Mixed | 826 | -1.41 | **<.001** |
| Outside Colombo | 221 | -3.13 | **<.001** |
| Financial strain, base = Living comfortably | 3933 |  |  |
| Doing alright | 365 | -1.52 | **<.001** |
| Just about getting by | 2616 | -2.90 | **<.001** |
| Difficult to make ends meet | 284 | -4.79 | **<.001** |
| V difficult to make ends meet | 129 | -5.93 | **<.001** |
| Education, base = No education | 47 |  |  |
| Grade 1-5 | 274 | 0.68 | .533 |
| Grade 6-O/Ls | 1757 | 1.40 | .159 |
| Passed O/Ls | 632 | 2.57 | .011 |
| Up to/Passed A/Ls | 929 | 3.45 | **.001** |
| University or higher | 276 | 2.84 | .009 |
| Other | 15 | 2.41 | .166 |
| Religion, base=Buddism | 3401 |  |  |
| Hindu | 64 | 1.47 | 0.087 |
| Islam | 159 | 2.16 | **<.001** |
| Christian | 309 | -.441 | 0.346 |
| Employment, base = Not in employment (incl. students) | 1758 |  |  |
| Employed | 2207 | 0.40 | .084 |
|  |  |  |  |

| **Deep-fried foods** |  |
| --- | --- |

| Age (Linear) | 3933 | -0.05 | **<.001** |
| --- | --- | --- | --- |
| Age (quartiles) base= 19-31 years | 1028 |  |  |
| 32-41 years | 1017 | -.603 | **.**006 |
| 42-53 years | 964 | -1.43 | **.000** |
| 54-91 years | 925 | -1.98 | **.000** |
| Sex, base = Male | 1681 |  |  |
| Female | 2288 | -1.14 | **<.001** |
| Region, base = Urban | 2390 |  |  |
| Rural | 532 | -0.77 | **<.001** |
| Mixed | 826 | -0.93 | **<.001** |
| Outside Colombo | 221 | -1.14 | **<.001** |
| Financial strain, base = Living comfortably | 3933 |  |  |
| Doing alright | 365 | .36 | .044 |
| Just about getting by | 2616 | -0.34 | .081 |
| Difficult to make ends meet | 284 | -0.52 | .024 |
| V difficult to make ends meet ***Became significant when outlier excluded: *b*=-0.90, *p*<.001** | 129 | -0.48 | .319 |
| Education, base = No education | 47 |  |  |
| Grade 1-5 | 274 | -0.22 | .650 |
| Grade 6-O/Ls | 1757 | 0.14 | .754 |
| Passed O/Ls | 632 | 0.02 | .966 |
| Up to/Passed A/Ls | 929 | 0.25 | .581 |
| University or higher | 276 | 0.86 | .123 |
| Other | 15 | -0.66 | .276 |
| Religion, base=Buddism | 3401 |  |  |
| Hindu | 64 | .29 | .614 |
| Islam | 159 | .56 | .120 |
| Christian | 309 | -.11 | .620 |
| Employment, base = Not in employment (incl. students) | 1758 |  |  |
| Employed | 2207 | 1.05 | **<.001** |
|  |  |  |  |

| **Salty Snacks** |  |
| --- | --- |

| Age (Linear) | 3933 | -0.03 | **<.001** |
| --- | --- | --- | --- |
| Age (quartiles) base= 19-31 years | 1028 |  |  |
| 32-41 years | 1017 | -.58 | **0.002** |
| 42-53 years | 964 | -1.116 | **0.00** |
| 54-91 years | 925 | -1.34 | **0.00** |
| Sex, base = Male | 1681 |  |  |
| Female | 2288 | -0.36 | .003 |
| Region, base = Urban | 2390 |  |  |
| Rural | 532 | 1.66 | **<.001** |
| Mixed | 826 | -0.76 | **<.001** |
| Outside Colombo | 221 | -0.80 | **<.001** |
| Financial strain, base = Living comfortably | 3933 |  |  |
| Doing alright | 365 | 0.75 | **<.001** |
| Just about getting by | 2616 | -0.09 | .646 |
| Difficult to make ends meet | 284 | -0.68 | **<.001** |
| V difficult to make ends meet | 129 | -0.18 | .592 |
| Education, base = No education | 47 |  |  |
| Grade 1-5 | 274 | -0.26 | .590 |
| Grade 6-O/Ls | 1757 | 0.37 | .407 |
| Passed O/Ls | 632 | -0.42 | .358 |
| Up to/Passed A/Ls | 929 | 0.11 | .803 |
| University or higher | 276 | 0.33 | .520 |
| Other | 15 | 0.79 | .369 |
| Religion, base=Buddism | 3401 |  |  |
| Hindu | 64 | -.45 | 0.346 |
| Islam | 159 | -.11 | 0.733 |
| Christian | 309 | -.27 | 0.235 |
| Employment, base = Not in employment (incl. students) | 1758 |  |  |
| Employed | 2207 | 0.31 | .012 |
|  |  |  |  |

| **Dessert/sweet snacks** |  |
| --- | --- |

| Age (Linear) | 3933 | -0.05 | **<.001** |
| --- | --- | --- | --- |
| Age (quartiles) base= 19-31 years | 1028 |  |  |
| 32-41 years | 1017 | -.78 | 0.046 |
| 42-53 years | 964 | -1.90 | **<.001** |
| 54-91 years | 925 | -1.89 | **<.001** |
| Sex, base = Male | 1681 |  |  |
| Female | 2288 | -0.95 | **<.001** |
| Region, base = Urban | 2390 |  |  |
| Rural | 532 | 4.51 | **<.001** |
| Mixed | 826 | 1.32 | **<.001** |
| Outside Colombo | 221 | -1.11 | .016 |
| Financial strain, base = Living comfortably | 3933 |  |  |
| Doing alright | 365 | 0.92 | .013 |
| Just about getting by | 2616 | -2.46 | **<.001** |
| Difficult to make ends meet | 284 | -3.81 | **<.001** |
| V difficult to make ends meet | 129 | -4.20 | **<.001** |
| Education, base = No education | 47 |  |  |
| Grade 1-5 | 274 | 0.33 | .753 |
| Grade 6-O/Ls | 1757 | 1.11 | .249 |
| Passed O/Ls | 632 | 0.27 | .779 |
| Up to/Passed A/Ls | 929 | 1.27 | .193 |
| University or higher | 276 | 1.21 | .246 |
| Other | 15 | 3.16 | .224 |
| Religion, base=Buddism | 3401 |  |  |
| Hindu | 64 | -.51 | 0.568 |
| Islam | 159 | -.05 | 0.926 |
| Christian | 309 | -1.65 | **0.000** |
| Employment, base = Not in employment (incl. students) | 1758 |  |  |
| Employed | 2207 | 1.14 | **<.001** |
|  |  |  |  |

| **Nuts/Seeds** |  |
| --- | --- |

| Age (Linear) | 3933 | -0.01 | **<.001** |
| --- | --- | --- | --- |
| Age (quartiles) base= 19-31 years | 1028 |  |  |
| 32-41 years | 1017 | 0.022 | **0.786** |
| 42-53 years | 964 | -.167 | **0.035** |
| 54-91 years | 925 | -.413 | **0.00** |
| Sex, base = Male | 1681 |  |  |
| Female | 2288 | -0.06 | .287 |
| Region, base = Urban | 2390 |  |  |
| Rural | 532 | 0.31 | **<.001** |
| Mixed | 826 | -0.06 | .293 |
| Outside Colombo | 221 | 0.04 | .713 |
| Financial strain, base = Living comfortably | 3933 |  |  |
| Doing alright | 365 | 0.19 | .010 |
| Just about getting by | 2616 | -0.01 | .906 |
| Difficult to make ends meet | 284 | -0.07 | .402 |
| V difficult to make ends meet | 129 | -0.13 | .288 |
| Education, base = No education | 47 |  |  |
| Grade 1-5 | 274 | -0.51 | .263 |
| Grade 6-O/Ls | 1757 | -0.12 | .797 |
| Passed O/Ls | 632 | -0.29 | .515 |
| Up to/Passed A/Ls | 929 | -0.20 | .659 |
| University or higher | 276 | -0.33 | .473 |
| Other | 15 | 0.13 | .830 |
| Religion, base=Buddism | 3401 |  |  |
| Hindu | 64 | -.29 | .106 |
| Islam | 159 | -.11 | 0.324 |
| Christian | 309 | -.25 | **0.007** |
| Employment, base = Not in employment (incl. students) | 1758 |  |  |
| Employed | 2207 | 0.15 | .003 |
|  |  |  |  |

| **Fruits** |  |
| --- | --- |

| Age (linear) | 3933 | 0.02 | .002 |
| --- | --- | --- | --- |
| Age (quartiles) base= 19-31 years | 1028 |  |  |
| 32-41 years | 1017 | .43 | 0.154 |
| 42-53 years | 964 | .32 | 0.273 |
| 54-91 years | 925 | 1.02 | 0.001 |
| Sex, base = Male | 1681 |  |  |
| Female | 2288 | 0.01 | .975 |
| Region, base = Urban | 2390 |  |  |
| Rural | 532 | -2.64 | **<.001** |
| Mixed | 826 | -0.95 | **<.001** |
| Outside Colombo | 221 | -1.18 | .004 |
| Financial strain, base = Living comfortably | 3933 |  |  |
| Doing alright | 365 | -2.39 | **<.001** |
| Just about getting by | 2616 | -5.08 | **<.001** |
| Difficult to make ends meet | 284 | -5.51 | **<.001** |
| V difficult to make ends meet | 129 | -7.69 | **<.001** |
| Education, base = No education | 47 |  |  |
| Grade 1-5 | 274 | -0.04 | .962 |
| Grade 6-O/Ls | 1757 | 1.33 | .062 |
| Passed O/Ls | 632 | 3.02 | **<.001** |
| Up to/Passed A/Ls | 929 | 3.25 | **<.001** |
| University or higher | 276 | 3.39 | **<.001** |
| Other | 15 | 2.24 | .112 |
| Religion, base=Buddism | 3401 |  |  |
| Hindu | 64 | -.48 | .547 |
| Islam | 159 | -.14 | .799 |
| Christian | 309 | -.94 | **.010** |
| Employment, base = Not in employment (incl. students) | 1758 |  |  |
| Employed | 2207 | -0.37 | .063 |
|  |  |  |  |

| **Leafy greens** |  |
| --- | --- |

| Age (Linear) | 3933 | 0.03 | **<.001** |
| --- | --- | --- | --- |
| Age (quartiles) base= 19-31 years | 1028 |  |  |
| 32-41 years | 1017 | .49 | **0.057** |
| 42-53 years | 964 | .67 | **0.012** |
| 54-91 years | 925 | 1.23 | **<.001** |
| Sex, base = Male | 1681 |  |  |
| Female | 2288 | -0.18 | .309 |
| Region, base = Urban | 2390 |  |  |
| Rural | 532 | -0.78 | .001 |
| Mixed | 826 | -0.26 | .243 |
| Outside Colombo | 221 | 0.26 | .503 |
| Financial strain, base = Living comfortably | 3933 |  |  |
| Doing alright | 365 | 0.68 | .008 |
| Just about getting by | 2616 | -1.12 | **<.001** |
| Difficult to make ends meet | 284 | -1.16 | .002 |
| V difficult to make ends meet | 129 | -2.65 | **<.001** |
| Education, base = No education | 47 |  |  |
| Grade 1-5 | 274 | -0.33 | .681 |
| Grade 6-O/Ls | 1757 | 0.37 | .629 |
| Passed O/Ls | 632 | 0.35 | .653 |
| Up to/Passed A/Ls | 929 | 0.59 | .442 |
| University or higher | 276 | 1.00 | .230 |
| Other | 15 | 0.54 | .752 |
| Religion, base=Buddism | 3401 |  |  |
| Hindu | 64 | -1.51 | 0.006 |
| Islam | 159 | -2.54 | **<.001** |
| Christian | 309 | -.475 | .174 |
| Employment, base = Not in employment (incl. students) | 1758 |  |  |
| Employed | 2207 | -0.20 | .266 |
|  |  |  |  |

| **Vegetables** |  |
| --- | --- |

| Age (Linear) | 3933 | 0.01 | .092 |
| --- | --- | --- | --- |
| Age (quartiles) base= 19-31 years | 1028 |  |  |
| 32-41 years | 1017 | .26 | .36 |
| 42-53 years | 964 | .72 | .017 |
| 54-91 years | 925 | .39 | .20 |
| Sex, base = Male | 1681 |  |  |
| Female | 2288 | -0.29 | .160 |
| Region, base = Urban | 2390 |  |  |
| Rural | 532 | 1.30 | .002 |
| Mixed | 826 | 1.95 | **<.001** |
| Outside Colombo | 221 | 0.48 | .293 |
| Financial strain, base = Living comfortably | 3933 |  |  |
| Doing alright | 365 | -0.88 | .006 |
| Just about getting by | 2616 | -2.66 | **<.001** |
| Difficult to make ends meet | 284 | -3.79 | **<.001** |
| V difficult to make ends meet | 129 | -5.02 | **<.001** |
| Education, base = No education | 47 |  |  |
| Grade 1-5 | 274 | -0.36 | .759 |
| Grade 6-O/Ls | 1757 | 0.76 | .500 |
| Passed O/Ls | 632 | 1.30 | .252 |
| Up to/Passed A/Ls | 929 | 1.42 | .211 |
| University or higher | 276 | 1.09 | .357 |
| Other | 15 | 1.75 | .471 |
| Religion, base=Buddism | 3401 |  |  |
| Hindu | 64 | -1.30 | 0.05 |
| Islam | 159 | -3.15 | **<.001** |
| Christian | 309 | -1.87 | **<.001** |
| Employment, base = Not in employment (incl. students) | 1758 |  |  |
| Employed | 2207 | 0.27 | .179 |

Note: Bonferroni corrected p-value = .0002. β=standardised beta coefficient.

**Table S3. Constrained correlation model (equal means and variances across birth-order, zygosity group, and sex) estimates for height and weight**

|  | **MZM** | **DZM** | **MZF** | **DZF** | **DZOS** |
| --- | --- | --- | --- | --- | --- |
| **Height** | **0.88**  **[0.86 - 0.90]** | **0.47**  **[0.33 - 0.58]** | **0.91**  **[0.89 - 0.92]** | **0.68**  **[0.60 - 0.74]** | **0.39**  **[0.29 - 0.47]** |
| **Weight** | **0.77**  **[0.72 - 0.81]** | **0.48**  **[0.33 - 0.59]** | **0.84**  **[0.80 - 0.86]** | **0.60**  **[0.51 - 0.67]** | **0.32**  **[0.22 - 0.41]** |

**Table S4. Best-fitting univariate ACE model estimates for height and weight**

|  | **A** | **C** | **E** |
| --- | --- | --- | --- |
| **Height**  (Quantitative Heterogeneity ACE) |  |  |  |
| **Male** | **0.88 [0.82 - 0.91]** | 0.01 [0.00 - 0.06] | **0.11 [0.09 - 0.13]** |
| **Female** | **0.48 [0.36 - 0.63]** | **0.42 [0.27 - 0.55]** | **0.10 [0.08 - 0.11]** |
| **Weight**  (Quantitative Heterogeneity ACE) |  |  |  |
| **Male** | **0.78 [0.72 - 0.82]** | 0.00 [0.00 - 0.05] | **0.21 [0.18 - 0.26]** |
| **Female** | **0.48 [0.32 - 0.67]** | **0.35 [0.16 - 0.50]** | **0.17 [0.15 - 0.21]** |

**Table S5. Univariate Model-Fitting Results for Frequency of Food Groups (sex & age adjusted and transformed when needed)**

| Meat | | | | | | | | |  |
| --- | --- | --- | --- | --- | --- | --- | --- | --- | --- |
| Base | Comparison | ep | LL | df | AIC | DiffLL | Diffdf | p-value |  |
| Full-HetACEc | - | 9 | 10879.33 | 3846 | 3187.33 | - | - | - |  |
| Full-HetACEc | **HetACE** | 8 | 10879.46 | 3847 | 3185.46 | 0.14 | 1 | 0.713 |  |
| Full-HetACEa | - | 9 | 10879.33 | 3846 | 3187.46 | - | - | - |  |
| Full-HetACEa | HetACE | 8 | 10879.46 | 3847 | 3187.33 | 0.00 | 1 | 1 |  |
| HetACE | HomACE | 5 | 11213.74 | 3850 | 3513.74 | 334.27 | 3 | **0.000** |  |
| HetACE | ScACE | 6 | 10888.87 | 3849 | 3190.87 | 9.41 | 2 | **0.009** |  |
| Fish |  |  |  |  |  |  |  |  |  |
| Base | Comparison | ep | LL | df | AIC | DiffLL | Diffdf | p-value |  |
| Full-HetACEc | - | 9 | 6869.67 | 3846 | -822.33 | - | - | - |  |
| Full-HetACEc | HetACE | 8 | 6869.67 | 3847 | -824.33 | 0.00 | 1 | 1 |  |
| Full-HetACEa | - | 9 | 6869.67 | 3846 | -822.33 | - | - | - |  |
| Full-HetACEa | HetACE | 8 | 6869.67 | 3847 | -824.33 | 0.00 | 1 | 1 |  |
| HetACE | **HomACE** | 5 | 6869.88 | 3850 | -830.12 | 0.21 | 3 | 0.976 |  |
| HetACE | ScACE | 6 | 6869.84 | 3849 | -828.16 | 0.17 | 2 | 0.917 |  |
| Eggs |  |  |  |  |  |  |  |  |  |
| Base | Comparison | ep | LL | df | AIC | DiffLL | Diffdf | p-value |  |
| Full-HetACEc | - | 9 | 258.74 | 3846 | -7433.26 | - | - | - |  |
| Full-HetACEc | HetACE | 8 | 258.74 | 3847 | -7435.26 | 0.00 | 1 | 1 |  |
| Full-HetACEa | - | 9 | 258.74 | 3846 | -7433.26 | - | - | - |  |
| Full-HetACEa | HetACE | 8 | 258.74 | 3847 | -7435.26 | 0.00 | 1 | 1 |  |
| HetACE | HomACE | 5 | 298.47 | 3850 | -7401.53 | 39.73 | 3 | **0.000** |  |
| HetACE | **ScACE** | 6 | 259.19 | 3849 | -7438.81 | 0.45 | 2 | 0.799 |  |
| Grains |  |  |  |  |  |  |  |  |  |
| Base | Comparison | ep | LL | df | AIC | DiffLL | Diffdf | p-value |  |
| Full-HetACEc | - | 9 | 2832.11 | 3846 | -4859.89 | - | - | - |  |
| Full-HetACEc | HetACE | 8 | 2832.11 | 3847 | -4861.89 | -0.00 | 1 | 1 |  |
| Full-HetACEa | - | 9 | 2832.11 | 3846 | -4859.89 | - | - | - |  |
| Full-HetACEa | HetACE | 8 | 2832.11 | 3847 | -4861.89 | -0.00 | 1 | 1 |  |
| HetACE | HomACE | 5 | 2835.54 | 3850 | -4864.46 | 3.44 | 3 | 0.329 |  |
| HetACE | **ScACE** | 6 | 2832.98 | 3849 | -4865.02 | 0.88 | 2 | 0.646 |  |
| Rice |  | | | | | | | |  |
| Base | Comparison | ep | LL | df | AIC | DiffLL | Diffdf | p-value |  |
| Full-HetACEc | - | 9 | 23449.97 | 3845 | 15759.97 | - | - | - |  |
| Full-HetACEc | HetACE | 8 | 23449.97 | 3846 | 15757.97 | -0.00 | 1 | 1 |  |
| Full-HetACEa | - | 9 | 23449.97 | 3845 | 15759.97 | - | - | - |  |
| Full-HetACEa | HetACE | 8 | 23449.97 | 3846 | 15757.97 | -0.00 | 1 | 1 |  |
| HetACE | HomACE | 5 | 23457.31 | 3849 | 15759.31 | 7.34 | 3 | 0.062 |  |
| HetACE | **ScACE** | 6 | 23457.06 | 3848 | 15761.06 | 7.09 | 2 | **0.029** |  |
| Flour |  | | | | | | | |  |
| Base | Comparison | ep | LL | df | AIC | DiffLL | Diffdf | p-value |  |
| Full-HetACEc | - | 9 | 2639.23 | 3843 | -5046.77 | - | - | - |  |
| Full-HetACEc | HetACE | 8 | 2639.23 | 3844 | -5048.77 | 0.00 | 1 | 1 |  |
| Full-HetACEa | - | 9 | 2639.23 | 3843 | -5046.77 | - | - | - |  |
| Full-HetACEa | HetACE | 8 | 2639.23 | 3844 | -5048.77 | 0.00 | 1 | 1 |  |
| HetACE | **HomACE** | 5 | 2640.22 | 3847 | -5053.78 | 0.99 | 3 | 0.803 |  |
| HetACE | ScACE | 6 | 2639.73 | 3846 | -5052.27 | 0.51 | 2 | 0.776 |  |
| Dairy | | | | | | | | |  |
| Base | Comparison | ep | LL | df | AIC | DiffLL | Diffdf | p-value |  |
| Full-HetACEc | - | 9 | 25791.23 | 3844 | 18103.23 | - | - | - |  |
| Full-HetACEc | HetACE | 8 | 25791.23 | 3845 | 18101.23 | 0.00 | 1 | 1 |  |
| Full-HetACEa | - | 9 | 25791.23 | 3844 | 18103.23 | - | - | - |  |
| Full-HetACEa | HetACE | 8 | 25791.23 | 3845 | 18101.23 | 0.00 | 1 | 1 |  |
| HetACE | HomACE | 5 | 25805.43 | 3848 | 18109.43 | 14.20 | 3 | **0.003** |  |
| HetACE | **ScACE** | 6 | 25795.66 | 3847 | 18101.66 | 4.43 | 2 | 0.109 |  |
| Deep Fried Food | | | | | | | | | |
| Base | Comparison | ep | LL | df | AIC | DiffLL | Diffdf | p-value |  |
| Full-HetACEc | - | 9 | 180.81 | 3845 | -7509.19 | - | - | - |  |
| Full-HetACEc | HetACE | 8 | 180.81 | 3846 | -7511.19 | 0.00 | 1 | 1 |  |
| Full-HetACEa | - | 9 | 180.81 | 3845 | -7509.19 | - | - | - |  |
| Full-HetACEa | HetACE | 8 | 180.81 | 3846 | -7511.19 | 0.00 | 1 | 1 |  |
| HetACE | HomACE | 5 | 349.73 | 3849 | -7348.27 | 168.92 | 3 | **0.000** |  |
| HetACE | **ScACE** | 6 | 184.58 | 3848 | -7511.42 | 3.76 | 2 | 0.152 |  |
| Salty Snacks | | | | | | | | |  |
| Base | Comparison | ep | LL | df | AIC | DiffLL | Diffdf | p-value |  |
| Full-HetACEc | - | 9 | 69.26 | 3845 | -7620.74 | - | - | - |  |
| Full-HetACEc | HetACE | 8 | 69.26 | 3846 | -7622.74 | 0.00 | 1 | 1 |  |
| Full-HetACEa | - | 9 | 69.26 | 3845 | -7620.74 | - | - | - |  |
| Full-HetACEa | HetACE | 8 | 69.26 | 3846 | -7622.74 | 0.00 | 1 | 1 |  |
| HetACE | HomACE | 5 | 84.15 | 3849 | -7613.85 | 14.89 | 3 | **0.002** |  |
| HetACE | **ScACE** | 6 | 71.24 | 3848 | -7624.76 | 1.98 | 2 | 0.372 |  |
| Desserts | | | | | | | | |  |
| Base | Comparison | ep | LL | df | AIC | DiffLL | Diffdf | p-value |  |
| Full-HetACEc | - | 9 | 3573.53 | 3845 | -4116.47 | - | - | - |  |
| Full-HetACEc | HetACE | 8 | 3573.53 | 3846 | -4118.47 | 0.00 | 1 | 1 |  |
| Full-HetACEa | - | 9 | 3573.53 | 3845 | -4116.47 | - | - | - |  |
| Full-HetACEa | HetACE | 8 | 3573.53 | 3846 | -4118.47 | 0.00 | 1 | 1 |  |
| HetACE | HomACE | 5 | 3599.80 | 3849 | -4098.20 | 26.28 | 3 | **0.000** |  |
| HetACE | **ScACE** | 6 | 3575.09 | 3848 | -4120.91 | 1.56 | 2 | 0.458 |  |
| Nuts & Seeds | | | | | | | | |  |
| Base | Comparison | ep | LL | df | AIC | DiffLL | Diffdf | p-value |  |
| Full-HetACEc | - | 9 | 11348.78 | 3844 | 3660.78 | - | - | - |  |
| Full-HetACEc | HetACE | 8 | 11351.06 | 3845 | 3661.06 | 2.28 | 1 | 0.131 |  |
| Full-HetACEa | - | 9 | 11351.06 | 3844 | 3663.06 | - | - | - |  |
| Full-HetACEa | HetACE | 8 | 11351.06 | 3845 | 3661.06 | 0.00 | 1 | 0.999 |  |
| HetACE | HomACE | 5 | 11361.37 | 3848 | 3665.37 | 10.03 | 3 | **0.016** |  |
| HetACE | **ScACE** | 6 | 11354.87 | 3847 | 3660.86 | 3.81 | 2 | 0.149 |  |
| Fruit |  |  |  |  |  |  |  |  |  |
| Base | Comparison | ep | LL | df | AIC | DiffLL | Diffdf | p-value |  |
| Full-HetACEc | - | 9 | 2616.28 | 3845 | -5073.72 | - | - | - |  |
| Full-HetACEc | HetACE | 8 | 2616.28 | 3846 | -5075.72 | 0.00 | 1 | 1 |  |
| Full-HetACEa | - | 9 | 2616.28 | 3845 | -5073.72 | - | - | - |  |
| Full-HetACEa | HetACE | 8 | 2616.28 | 3846 | -5075.72 | 0.00 | 1 | 1 |  |
| HetACE | **HomACE** | 5 | 2617.11 | 3849 | -5080.89 | 0.82 | 3 | 0.844 |  |
| HetACE | ScACE | 6 | 2617.04 | 3848 | -5078.96 | 0.76 | 2 | 0.685 |  |
| Leafy Greens | | | | | | | | |  |
| Base | Comparison | ep | LL | df | AIC | DiffLL | Diffdf | p-value |  |
| Full-HetACEc | - | 9 | 5217.40 | 3846 | -2474.60 | - | - | - |  |
| Full-HetACEc | HetACE | 8 | 5217.40 | 3847 | -2476.60 | 0.00 | 1 | 1 |  |
| Full-HetACEa | - | 9 | 5217.40 | 3846 | -2474.6 | - | - | - |  |
| Full-HetACEa | HetACE | 8 | 5217.40 | 3847 | -2476.60 | 0.00 | 1 | 1 |  |
| HetACE | **HomACE** | 5 | 5219.67 | 3850 | -2480.33 | 2.26 | 3 | 0.519 |  |
| HetACE | ScACE | 6 | 5219.55 | 3849 | -2478.45 | 2.15 | 2 | 0.342 |  |
| Vegetables | | | | | | | | | |
| Base | Comparison | ep | LL | df | AIC | DiffLL | Diffdf | p-value |  |
| Full-HetACEc | - | 9 | 11494.93 | 3844 | 3806.925 | - | - | - |  |
| Full-HetACEc | **HetACE** | 8 | 11498.65 | 3845 | 3808.653 | 3.73 | 1 | 0.054 |  |
| Full-HetACEa | - | 9 | 11498.65 | 3844 | 3810.653 | - | - | - |  |
| Full-HetACEa | HetACE | 8 | 11498.65 | 3845 | 3808.653 | 0.00 | 1 | 1 |  |
| HetACE | HomACE | 5 | 11509.68 | 3848 | 3813.685 | 11.03 | 3 | **0.011** |  |
| HetACE | ScACE | 6 | 11507.46 | 3847 | 3813.457 | 8.80 | 2 | **0.012** |  |

Note: LL = -2 Log-Likelihood of the data under the model, ep = number of estimated parameters in the model, df = Degree-of-Freedom, AIC = Akaike’s Information Criteria (LL minus 2*df). Likelihood Ratio testing: evaluating the differences in LL and df (DiffLL and Diffdf) of the base and comparison model, with the associated p-value. Best-fitting models (bold) are based on likelihood ratio testing (and lowest AIC) and explained below by means of the ratios of the MZ and DZ twin correlations in males and females and the DZ opposite-sex correlations (reported in Table 3 of the manuscript).

**Meat and Vegetables:** Qualitative sex differences are not indicated because, although rDZO is significant, it is not significantly smaller than rDZM nor rDZF; rMZM overlaps with rDZM and rMZF overlaps with rDZF (or is slightly lower); The correlation ratios suggest ACE for males and CE for females and therefore the Heterogeneity model to fit best.

**Fish:** Qualitative sex differences are not indicated because, although rDZO is significant, it is not significantly smaller than rDZM nor rDZF; rMZM overlaps with rDZM, and rMZF overlaps with rDZF; The correlation ratios suggest CE for both males and females and therefore the Homogeneity model to fit best.

**Flour & Leafy greens:** Qualitative sex differences are not indicated because, although rDZO is significant, it is not significantly smaller than rDZM nor rDZF; rMZM overlaps with rDZM and rMZF overlaps with rDZF The overall lower DZ correlation is low due to the very small rDZO. The correlation ratios suggest ACE for both males and females and the Homogeneity model to fit best.

**Fruits:** Qualitative sex differences are not indicated because, although rDZO is significant, it is not significantly smaller than rDZM nor rDZF; rMZM does not overlap with rDZM and rMZF does not overlap with rDZF (due to the tighter CI when including rDZO). The correlation ratios suggest ACE for both males and females and the Homogeneity model to fit best.

**Eggs, Grains, Rice, Dairy, Deep Fried Foods:** Qualitative sex differences are not indicated because, although rDZO is significant, it is not significantly smaller than rDZM nor rDZF; rMZM overlaps with rDZM, and rMZF overlaps with rDZF (due to the higher overall DZ correlation when including rDZO). The correlation ratios suggest CE for both males and females, although there is a difference in absolute variance which needs to be accounted for. Therefore, the no-sex-difference Variance Inequality model fits best.

**Salty Snacks, Desserts/Sweet Snacks:** Qualitative sex differences are not indicated because, although rDZO is significant, it is not significantly smaller than rDZM nor rDZF; rMZM overlaps with rDZM, and rMZF overlaps with rDZF. The correlation ratios suggest CE for both males and females, although there is a difference in absolute variance which needs to be accounted for. Therefore, the no-sex-difference Variance Inequality model fits best. The small magnitude of the correlations, however, might not give enough power to show significant familial components (A or C).

**Nuts/Seeds:** Qualitative sex differences are not indicated because, although rDZO is non-significant and not significantly smaller than rDZM nor rDZF; rMZM overlaps with rDZM, and rMZF overlaps with rDZF. The correlation ratios suggest CE for both males and females, although there is a difference in absolute variance which needs to be accounted for. Therefore, the no-sex-difference Variance Inequality model fits best. The small magnitude of the correlations, however, might not give enough power to show significant familial components (A or C).

**Table S6. Univariate Model-Fitting Results for the Cardio Metabolic Risk Variables (sex & age adjusted and transformed when needed)**

| Waist Circumference | | | | | | | | |  |
| --- | --- | --- | --- | --- | --- | --- | --- | --- | --- |
| Base | Comparison | ep | LL | df | AIC | DiffLL | Diffdf | p-value |  |
| Full-HetACEc | - | 9 | 26869.15 | 3561 | 19747.15 | - | - | - |  |
| Full-HetACEc | HetACE | 8 | 26874.45 | 3562 | 19750.45 | 5.30 | 1 | **0.021** |  |
| Full-HetACEa | - | 9 | 26869.15 | 3561 | 19747.15 | - | - | - |  |
| Full-HetACEa | HetACE | 8 | 26874.45 | 3562 | 19750.45 | 5.30 | 1 | **0.021** |  |
| HetACE | HomACE | 5 | 26900.95 | 3565 | 19770.95 | 26.50 | 3 | **0.000** |  |
| HetACE | ScACE | 6 | 26890.73 | 3564 | 19762.73 | 16.29 | 2 | **0.000** |  |
| BMI |  |  |  |  |  |  |  |  |  |
| Base | Comparison | ep | LL | df | AIC | DiffLL | Diffdf | p-value |  |
| Full-HetACEc | - | 9 | 20134.75 | 3554 | 13026.75 | - | - | - |  |
| Full-HetACEc | HetACE | 8 | 20140.06 | 3555 | 13030.06 | 5.31 | 1 | **0.021** |  |
| Full-HetACEa | - | 9 | 20134.75 | 3554 | 13026.75 | - | - | - |  |
| Full-HetACEa | HetACE | 8 | 20140.06 | 3555 | 13030.06 | 5.31 | 1 | **0.021** |  |
| HetACE | HomACE | 5 | 20184.40 | 3558 | 13068.40 | 44.34 | 3 | **0.000** |  |
| HetACE | ScACE | 6 | 20153.31 | 3557 | 13039.31 | 13.25 | 2 | **0.001** |  |
| BP systolic |  |  |  |  |  |  |  |  |  |
| Base | Comparison | ep | LL | df | AIC | DiffLL | Diffdf | p-value |  |
| Full-HetACEc | - | 9 | 29840.29 | 3566 | 22708.29 | - | - | - |  |
| Full-HetACEc | **HetACE** | 8 | 29840.29 | 3567 | 22706.29 | 0.00 | 1 | 1 |  |
| Full-HetACEa | - | 9 | 29838.85 | 3566 | 22706.85 | - | - | - |  |
| Full-HetACEa | HetACE | 8 | 29840.29 | 3567 | 22706.29 | 1.43 | 1 | 0.231 |  |
| HetACE | HomACE | 5 | 29861.85 | 3570 | 22721.85 | 21.57 | 3 | **0.000** |  |
| HetACE | ScACE | 6 | 29859.43 | 3569 | 22721.43 | 19.14 | 2 | **0.000** |  |
| BP diastolic |  |  |  |  |  |  |  |  |  |
| Base | Comparison | ep | LL | df | AIC | DiffLL | Diffdf | p-value |  |
| Full-HetACEc | - | 9 | 26814.86 | 3566 | 19682.86 | - | - | - |  |
| Full-HetACEc | **HetACE** | 8 | 26814.86 | 3567 | 19680.86 | 0.00 | 1 | 1 |  |
| Full-HetACEa | - | 9 | 26813.27 | 3566 | 19681.27 | - | - | - |  |
| Full-HetACEa | HetACE | 8 | 26814.86 | 3567 | 19680.86 | 1.59 | 1 | 0.207 |  |
| HetACE | HomACE | 5 | 26843.47 | 3570 | 19703.47 | 28.61 | 3 | **0.000** |  |
| HetACE | ScACE | 6 | 26837.28 | 3569 | 19699.28 | 22.42 | 2 | **0.000** |  |
| Triglycerides | **outliers included** | | | | | | | |  |
| Base | Comparison | ep | LL | df | AIC | DiffLL | Diffdf | p-value |  |
| Full-HetACEc | - | 9 | 8544.03 | 3373 | 1798.03 | - | - | - |  |
| Full-HetACEc | HetACE | 8 | 8544.16 | 3374 | 1796.03 | 0.13 | 1 | 0.715 |  |
| Full-HetACEa | - | 9 | 8544.03 | 3373 | 1798.03 | - | - | - |  |
| Full-HetACEa | HetACE | 8 | 8544.16 | 3374 | 1796.03 | 0.13 | 1 | 0.715 |  |
| HetACE | HomACE | 5 | 8828.62 | 3377 | 2074.62 | 284.46 | 3 | **0.000** |  |
| HetACE | **ScACE** | 6 | 8546.82 | 3376 | 1794.82 | 2.66 | 2 | 0.264 |  |
| Triglycerides | **outliers excluded** | | | | | | | |  |
| Base | Comparison | ep | LL | df | AIC | DiffLL | Diffdf | p-value |  |
| Full-HetACEc | - | 9 | 9112.82 | 3310 | 2492.82 | - | - | - |  |
| Full-HetACEc | **HetACE** | 8 | 9112.82 | 3311 | 2492.82 | 0 | 1 | 1 |  |
| Full-HetACEa | - | 9 | 9112.77 | 3310 | 2492.77 |  |  |  |  |
| Full-HetACEa | HetACE | 8 | 9112.82 | 3311 | 2492.82 | .05 | 1 | 1 |  |
| HetACE | HomACE | 5 | 9270.61 | 3314 | 2642.61 | 157.79 | 3 | **0.000** |  |
| HetACE | ScACE | 6 | 9127.97 | 3313 | 2501.97 | 15.14 | 2 | **0.001** |  |
| HDL cholesterol | | | | | | | | |  |
| Base | Comparison | ep | LL | df | AIC | DiffLL | Diffdf | p-value |  |
| Full-HetACEc | - | 9 | 10696.9 | 3373 | 3950.9 | - | - | - |  |
| Full-HetACEc | HetACE | 8 | 10696.9 | 3374 | 3948.9 | 0.00 | 1 | 1 |  |
| Full-HetACEa | - | 9 | 10696.63 | 3373 | 3950.6 | - | - | - |  |
| Full-HetACEa | HetACE | 8 | 10696.9 | 3374 | 3948.9 | 0.27 | 1 | 0.606 |  |
| HetACE | **HomACE** | 5 | 10702.12 | 3377 | **3948.1** | 5.22 | 3 | 0.156 |  |
| HetACE | ScACE | 6 | 10701.55 | 3376 | 3949.6 | 4.65 | 2 | 0.097 |  |
| Fasting glucose (outliers included) | | | | | | | | | |
| Base | Comparison | ep | LL | df | AIC | DiffLL | Diffdf | p-value |  |
| Full-HetACEc | - | 9 | 11240.49 | 3372 | 4496.495 | - | - | - |  |
| Full-HetACEc | HetACE | 8 | 11240.49 | 3373 | 4494.495 | 0.00 | 1 | 1 |  |
| Full-HetACEa | - | 9 | 11221.39 | 3372 | 4477.386 | - | - | - |  |
| Full-HetACEa | HetACE | 8 | 11240.49 | 3373 | 4494.495 | 19.1 | 1 | **0.000** |  |
| HetACE | **HomACE** | 5 | 11252.10 | 3376 | 4500.103 | 49.38 | 3 | **0.000** |  |
| HetACE | ScACE | 6 | 11285.28 | 3375 | 4535.281 | 53.30 | 2 | **0.000** |  |
| Fasting glucose (outliers excluded) | | | | | | | | |  |
| Base | Comparison | ep | LL | df | AIC | DiffLL | Diffdf | p-value |  |
| Full-HetACEc | - | 9 | 8539.967 | 3281 | 1977.97 | - | - | - |  |
| Full-HetACEc | HetACE | 8 | 8539.967 | 3282 | 1975.97 | 0 | 1 | 1 |  |
| Full-HetACEa | - | 9 | 8539.967 | 3281 | 1977.97 | - | - | - |  |
| Full-HetACEa | HetACE | 8 | 8539.967 | 3282 | 1975.97 | 0 | 1 | 1 |  |
| HetACE | **HomACE** | 5 | 8541.878 | 3285 | 1971.88 | 1.91 | 3 | 0.591 |  |
| HetACE | ScACE | 6 | 8654.940 | 3284 | 2086.94 | 1.15 | 2 | 0.108 |  |
| Insulin (outliers included) | | | | | | | | |  |
| Base | Comparison | ep | LL | df | AIC | DiffLL | Diffdf | p-value |  |
| Full-HetACEc | - | 9 | 10047.05 | 3371 | 3305.048 | - | - | - |  |
| Full-HetACEc | **HetACE** | 8 | 10047.05 | 3372 | 3303.048 | 0.00 | 1 | 1 |  |
| Full-HetACEa | - | 9 | 10047.05 | 3371 | 3303.048 | - | - | - |  |
| Full-HetACEa | HetACE | 8 | 10047.05 | 3372 | 3303.048 | 0.00 | 1 | 1 |  |
| HetACE | HomACE | 5 | 10047.05 | 3375 | 3324.344 | 27.30 | 3 | **0.000** |  |
| HetACE | ScACE | 6 | 10053.78 | 3374 | 3305.780 | 6.73 | 2 | **0.035** |  |
| Insulin (outliers excluded) | | | | | | | | |  |
| Base | Comparison | ep | LL | df | AIC | DiffLL | Diffdf | p-value |  |
| Full-HetACEc | - | 9 | 9213.240 | 3353 | 2507.240 | - | - | - |  |
| Full-HetACEc | **HetACE** | 8 | 9213.240 | 3354 | 2505.240 | 0 | 1 | 1 |  |
| Full-HetACEa | - | 9 | 9213.240 | 3353 | 2507.240 | - | - | - |  |
| Full-HetACEa | HetACE | 8 | 9213.240 | 3354 | 2507.240 | 0 | 1 | 1 |  |
| HetACE | HomACE | 5 | 9245.731 | 3357 | 2531.731 | 32.49 | 3 | **0.000** |  |
| HetACE | ScACE | 6 | 9222.486 | 3356 | 2510.486 | 9.25 | 2 | **0.009** |  |

Note: LL = -2 Log-Likelihood of the data under the model, ep = number of estimated parameters in the model, df = Degree-of-Freedom, AIC = Akaike’s Information Criteria (LL minus 2*df). Likelihood Ratio testing: evaluating the differences in LL and df (DiffLL and Diffdf) of the base and comparison model, with the associated p-value. Best-fitting models (bold) are based on likelihood ratio testing (and lowest AIC) and explained below by means of the ratios of the MZ and DZ twin correlations in males and females and the DZ opposite-sex correlations (reported in Table 5 of the manuscript).

**WC and BMI:** Qualitative sex differences are significant because rDZO is significant and also significantly lower than rDZM and rDZF. Therefore, a model with either Qualitative A or C sex differences (Full-HetACEa or Full-HetACEc) shows the best fit, both producing the same estimates. rMZM does not overlap with rDZM and rMZF does not overlap with rDZF. The correlation ratios suggest ACE for males and females. The ACE parameters are estimated separately for males and females by default in this model.

**BP:** Qualitative sex differences are not indicated since rDZO ~ 0 and not significantly smaller than either rDZM and rDZF; For **BP systolic**, rMZM does not overlap with rDZM and rMZF does not overlap with rDZF. The correlation ratios suggest AE for males and females. For **BP diastolic**, rMZM does overlap with rDZM and rMZF does not overlap with rDZF. The correlation ratios suggest ACE for males and AE for females and therefore the Heterogeneity model to fit best.

**Triglycerides (outliers included):** Qualitative sex differences are not indicated because, although rDZO is significant, it is not significantly smaller than rDZM nor rDZF; rMZM overlaps with rDZM and rMZF does not overlap with rDZF. The correlation ratios suggest ACE for males and AE for females, although there is a difference in absolute variance which needs to be accounted for. Therefore, the no-sex-difference Variance Inequality model fits best.

**Triglycerides (outliers excluded):** Qualitative sex differences are not indicated because rDZO is not significant and not significantly smaller than rDZM and rDZF. rMZM overlaps with rDZM and rMZF does not overlap with rDZF. The correlation ratios suggest ACE for males and AE for females and the Heterogeneity model to fit best.

**HDL cholesterol:** Qualitative sex differences are not indicated because, although rDZO is significant, it is not significantly smaller than rDZM and rDZF. rMZM does not overlap with rDZM, rMZF does not overlap with rDZF, meaning no effects of C and since the correlation ratios are the same, it suggests the Homogeneity model to fit best.

**Fasting Glucose (outliers included):** In this case rDZO is significant but both same-sex DZ correlations are not. rMZM does not overlap with rDZM, rMZF does not overlap with rDZF, indicating AE for both males and females and the Homogeneity model to fit best. It is not clear why the Qualitative A model shows the best fit given the correlations. We therefore stick with reporting the Homogeneity model.

**Fasting Glucose (outliers excluded):** Qualitative sex differences are not indicated because, although rDZO is significant, it is not significantly smaller than rDZM and rDZF. rMZM does not overlap with rDZM, rMZF does not overlap with rDZF, suggesting AE for both males and females and the Homogeneity model to fit best.

**Insulin (outliers included and excluded):** Qualitative sex differences are not indicated because, although rDZO is significant, it is not significantly smaller than rDZM and rDZF. rMZM overlaps with rDZM and rMZF does not overlap with rDZF, indicating ACE for males and AE for females and the Heterogeneity model to fit best.

**Table S7. Pearson correlations between the Frequency of Food Group (Diet) variables and the Cardio Metabolic Risk Variables (with 95%CI).**

|  | Waist circumference | BMI | Systolic blood pressure | Diastolic blood pressure | Triglycerides (mmol/l) | Cholesterol (mmol/l) | Glucose (mmol/l) |
| --- | --- | --- | --- | --- | --- | --- | --- |
| 1. Meat | .03 (-.01, .06) | .03 (-.00, .06) | .00 (-.03, .03) | .01 (-.03, .04) | .03 (.00, .07) | .00 (-.03, .03) | .02 (-.01, .05) |
| 1. Fish | **.04** **(.01, .08)** | .03 (-.00, .06) | -.01 (-.03, .03) | .01 (-.02, .05) | -.02 (-.05, .01) | .02 (-.02, .05) | -.00 (-.04, .03) |
| 1. Eggs | -.03 (-.07, .00) | -.03 (-.06, .00) | .01 (-.03, .04) | -.01 (-.04, .03) | -.00 (-.04, .03) | .02 (-.01, .06) | -.00 (-.03, .03) |
| 1. Grains | .02 (-.01, .05) | -.01 (-.05, .02) | -.01 (-.04, .02) | -.01 (-.05, .02) | -.00 (-.04, .03) | -.03 (-.06, .01) | -.03 (-.06, .01) |
| 1. Rice | -.01 (-.05, .02) | **-.05 (-.08, -.01)** | -.01 (-.04, .02) | -.02 (-.05, .02) | -.03 (-.06, .00) | -.01 (-.05, .02) | **-.04** **(-.08, -.01)** |
| 1. Flour | .01 (-.03, .04) | .02 (-.01, .06) | .02 (-.02, .05) | .01 (-.02, .05) | .03 (-.01, .06) | .01 (-.02, .04) | -.01 (-.04, .03) |
| 1. Dairy Products | -.03 (-.06, .00) | **-.05 (-.08, -.02)** | -.03 (-.06, .00) | **-.04** (-.07, -.01) | -.01 (-.04, .03) | -.00 (-.04, .03) | **-.05** **(-.08, -.01)** |
| 1. Deep fried foods | .01 (-.02, .05) | .00 (-.03, .04) | .03 (.00, .07) | .02 (-.01, .05) | .03 (-.00, .06) | .02 (-.02, .05) | -.02 (-.05, .02) |
| 1. Salty Snacks | -.02 (-.05, .01) | -.02 (-.05, .01) | .01 (-.02, .04) | -.02 (-.05, .02) | -.01 (-.05, .02) | -.02 (-.06, .01) | -.03 (-.06, .01) |
| 1. Dessert/sweet snacks | .00 (-.03, .03) | -.00 (-.03, .03) | .00 (-.03, .03) | -.01 (-.05, .02) | -.04 (-.07, -.00) | .01 (-.02, .04) | **-.05** **(-.09, -.02)** |
| 1. Nuts/Seeds | -.04 (-.07, .00) | -.04 (-.07, .00) | -.00 (-.04, .03) | -.01 (-.04, .02) | .01 (-.03, .04) | -.01 (-.05, .02) | -.00 (-.04, .03) |
| 1. Fruits | **.06** **(.02, .09)** | **.04** **(.01, .07)** | -.01 (-.04, .02) | -.02 (-.05, .01) | .02 (-.01, .05) | -.01 (-.05, .02) | .02 (-.01, .05) |
| 1. Leafy greens | .01 (-.02, .05) | .01 (-.02, .04) | -.02 (-.05, .01) | -.02 (-.05, .01) | -.01 (-.04, .03) | .03 (-.00, .06) | -.01 (-.04, .02) |
| 1. Vegetables | -.01 (-.04, .03) | -.02 (-.06, .01) | -.02 (-.05, .02) | .00 (-.03, .03) | -.00 (-.04, .03) | .01 (-.03, .04) | -.01 (-.04, .02) |

**Figure S1**

**Figure S1: Best-fitting univariate ACE model estimates for FFQ food groups, weekly intake.**

**Figure S2**

**Figure S2: Best-fitting univariate ACE model estimates for cardiometabolic risk variables.**

Note: ^*1,*2,*3^ Outliers (> 3 SD) removed, n=65, 91, 18, respectively.

**Figure S3**

**
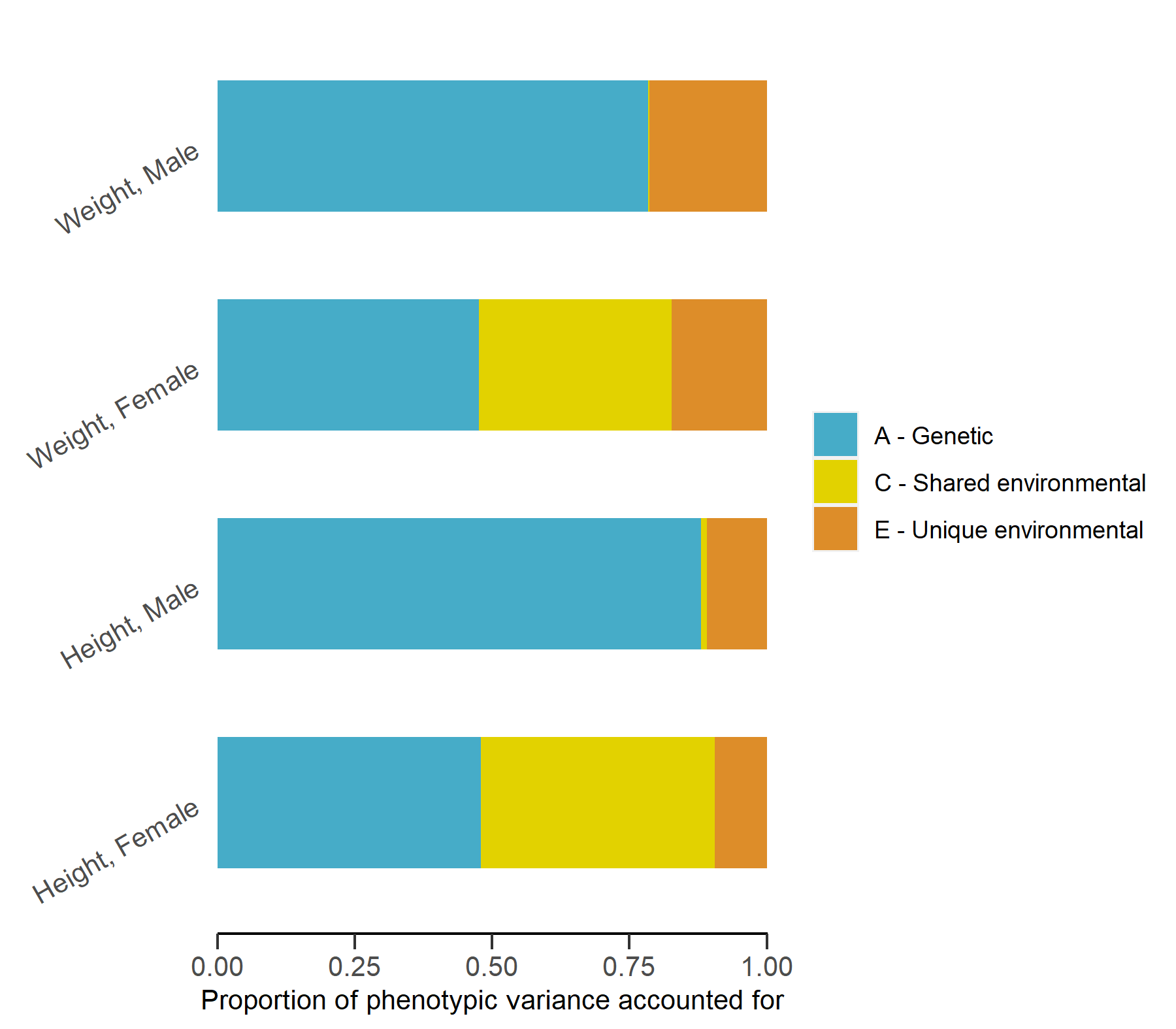
**

**Figure S1: Best-fitting ACE model estimates for Height and Weight**
